# An in vitro CRISPR screen of cell-free DNA identifies apoptosis as the primary mediator of cell-free DNA release

**DOI:** 10.1038/s42003-024-06129-1

**Published:** 2024-04-10

**Authors:** Brad. A. Davidson, Adam X. Miranda, Sarah C. Reed, Riley E. Bergman, Justin D. J. Kemp, Anvith P. Reddy, Morgan V. Pantone, Ethan K. Fox, R. Dixon Dorand, Paula J. Hurley, Sarah Croessmann, Ben Ho Park

**Affiliations:** 1 Division of Hematology, Oncology, Department of Medicine, Vanderbilt University Medical Center and the Vanderbilt-Ingram Cancer Center, Nashville, TN USA; 2 Medical Scientist Training Program, Vanderbilt University, Nashville, TN USA

**Keywords:** CRISPR-Cas9 genome editing, Apoptosis, Cancer genetics

## Abstract

Clinical circulating cell-free DNA (cfDNA) testing is now routine, however test accuracy remains limited. By understanding the life-cycle of cfDNA, we might identify opportunities to increase test performance. Here, we profile cfDNA release across a 24-cell line panel and utilize a cell-free CRISPR screen (cfCRISPR) to identify mediators of cfDNA release. Our panel outlines two distinct groups of cell lines: one which releases cfDNA fragmented similarly to clinical samples and purported as characteristic of apoptosis, and another which releases larger fragments associated with vesicular or necrotic DNA. Our cfCRISPR screens reveal that genes mediating cfDNA release are primarily involved with apoptosis, but also identify other subsets of genes such as RNA binding proteins as potential regulators of cfDNA release. We observe that both groups of cells lines identified primarily produce cfDNA through apoptosis. These results establish the utility of cfCRISPR, genetically validate apoptosis as a major mediator of DNA release in vitro, and implicate ways to improve cfDNA assays.

## Abstract (web summary)

A novel approach using whole-genome CRISPR-Cas9 screen indicates that apoptosis is the most important mediator of cfDNA release.

## Introduction

Cell-free DNA (cfDNA) is now routinely used in clinical medicine including obstetrics and transplant medicine, and has become a major diagnostic tool for clinical oncology^1–5^. However, a key issue with such tests is their suboptimal sensitivity due in large part to low and variable quantities of cfDNA in blood^6–10^. This is exemplified in clinical oncology, where the utility of circulating tumor DNA (ctDNA), which is cfDNA derived from cancer or precancerous cells, remains largely limited to metastatic disease to identify mutations/alterations that may have targeted therapies. In the clinically promising settings of cancer screening, early-stage disease diagnostics, and microscopic minimal residual disease the paucity of ctDNA and total cfDNA limits the sensitivity and negative predictive value of current “liquid biopsy” ctDNA tests. Most ongoing efforts to improve the sensitivity and specificity of ctDNA-based liquid biopsy have focused on including more or alternative analytes, and/or applying advanced sequencing techniques^11–14^. Few studies have leveraged the basic biogenesis, degradation, and elimination of total cfDNA and cancer-specific ctDNA to improve testing accuracy. ctDNA release is highly variable and levels range from undetectable to extremely high variant allele fractions relative to total cfDNA depending on cancer type, stage, and other unknown factors^15,16^. We reasoned that definitive knowledge of how cfDNA is released could be leveraged to augment the sensitivity of current liquid biopsies.

Apoptosis, necrosis, and active release through vesicular pathways are the most supported mechanisms of cfDNA and ctDNA release but remain heavily debated in the literature^17–19^. Based on the fragmentation pattern of cfDNA in blood, apoptosis has long been assumed to be the primary mechanism of cfDNA release from cells. cfDNA from both healthy controls and cancer patients is primarily found at ~167 bp in length with a ladder pattern that has been attributed to caspase-dependent apoptotic cleavage^20–23^. While the role of apoptosis has been implicated in multiple in vitro and in vivo studies^20,24,25^, other reports show a lack of correlation between apoptosis and cfDNA release^25–27^. In addition, ctDNA fragments are reported to be enriched at sizes significantly smaller and larger than this peak, suggesting ctDNA is further processed or released through additional pathways^28–30^. Other studies have proposed necrosis as a major source of cfDNA release corresponding instead to DNA fragments ~10,000 bp in size^20,25^. One such study showed that necrosis was correlated with ctDNA detection in a retrospective analysis of lung cancer patients^31^. Finally, vesicles released from cells are another widely reported mechanism associated with cfDNA release. While most studies observe vesicle-associated DNA is >1000 bp, studies have reached differing conclusions about the DNA content of various vesicle populations^32–39^. In 2019, Jeppesen et al. presented evidence that small extracellular vesicles do not contain DNA, contrary to previous studies^40^. These uncertainties regarding the origins of cfDNA underscore the need for additional research to gain new insights that could be translated for clinical care.

Here, we profiled in vitro cfDNA release in 24 human cell lines and observed that many cancer cells have cfDNA fragmentation patterns thought to be associated with non-apoptotic release mechanisms, with an enrichment of cfDNA at >1000 bp. However, using a cell-free DNA CRISPR screening strategy (cfCRISPR) in both a non-tumorigenic and cancer cell line, we identified genes involved in apoptotic processes as the primary effectors of cfDNA release. Two apoptotic regulatory genes (*FADD* and *BCL2L1*) and one RNA binding protein gene (*KHDRBS1*/Sam68) were further validated as mediators of cfDNA release. Additionally, cell lines could be induced to shed more cfDNA with the addition of Tumor Necrosis Factor-Related Apoptosis-Inducing Ligand (TRAIL). Modification of apoptosis through various methods led to changes in cfDNA release at large fragment sizes in all cell lines, even in those that initially display the expected 167 bp fragmentation pattern found in humans. This suggests that cfDNA released through the apoptotic pathway is not inherently cleaved to the “characteristic” apoptotic size of 167 bp, and that further cleavage likely occurs after cfDNA release. This study provides definitive genetic evidence that the apoptotic pathway is a major mediator of cfDNA release, establishes cfCRISPR as a valid tool for further genetic screens, and provides new insights to leverage this knowledge for clinical use.

## Results

### A panel of human cell lines reveals convergent cfDNA release kinetics and divergent fragmentation patterns

**Fig. 1:**
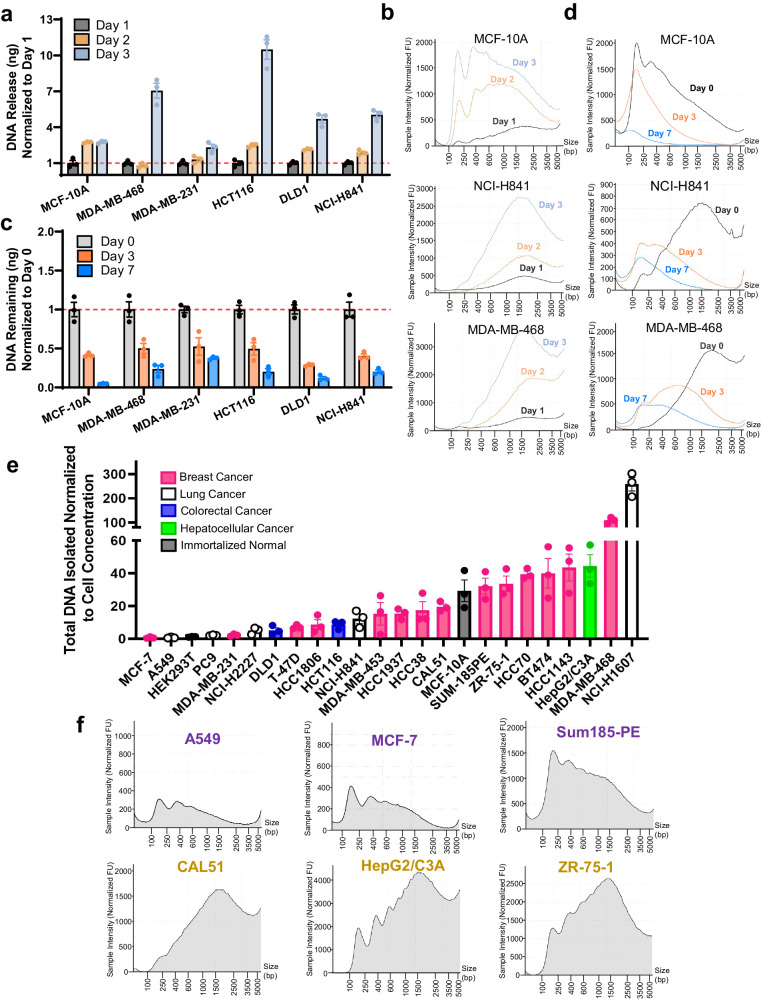
A panel of human cell lines reveals convergent cfDNA release kinetics and divergent fragmentation patterns. **a** Release of cfDNA in vitro over time. Data represent mean fold change ± SEM in absolute DNA release from Day 1 for each cell line, with *n* = 3 biologically independent samples. **b** Electropherograms of samples from **a**, assessing changes in cfDNA release with increased cell incubation periods, were individually run at least *n* = 3 times and representative traces are shown. **c** Degradation of in vitro cfDNA over time at physiologic temperatures. Data represent mean fold change ± SEM in absolute DNA quantity from Day 0 for each cell line, with *n* = 3 biologically independent samples. **d** Electropherograms of samples from c, assessing the degradation of cfDNA in culture media after removal from cells, were individually run at least *n* = 3 times and representative traces are shown. **e** Quantification of cfDNA release from cell lines in culture. Data represent mean fold change ± SEM in DNA release normalized to cell concentration at time of collection for each cell line, with *n* = 3 biologically independent samples. **f** Fragmentation patterns of selected cell lines from the cfDNA panel. Cell lines A549, MCF-7, and Sum185-PE (purple text) are representative of cell lines with a left skew, CAL51, HepG2/C3A, and ZR-75-1 (yellow text) are representative of cell lines with a right skew. Representative traces shown of at least *n* = 3 runs.

To accurately analyze mechanisms of cfDNA release in vitro, we initially assessed six human cell lines to develop a standardized cfDNA assay. Cells were grown without media changes for the indicated days, and designated replicates harvested for cfDNA at days 1, 2, and 3. Our isolation methodology specifically retains most large and small vesicular populations, allowing us to analyze their effects on cfDNA release. Despite reported discordance in cfDNA quantitation over time^24,25,27^, all cell lines demonstrated increases in the quantity of cfDNA over 3 days (Fig. 1a). Furthermore, in contrast to previously reported large shifts in fragmentation patterns, in some cases moving from small 167 bp fragments to large >1500 bp fragments over time^26^, we primarily observed an increase in the concentration of fragments at the same size that was most prevalent at previous time points (Fig. 1b). To characterize the contribution of DNA degradation in our system, media containing cfDNA was collected at day 3, incubated in new flasks without cells, and profiled 0, 3, and 7 days after removal. The half-life of cfDNA for all cell lines was approximately 3 days, with smaller fragments appearing over the course of the assay (Fig. 1c, d). These results suggest that DNA is degrading in vitro over several days and suggests that small fragments may be derived from degradation of larger cfDNA products after cellular release.

cfDNA release properties were then assessed in a larger panel of 24 human cell lines. This panel included nontumorigenic (*n* = 2), hepatocellular cancer (*n* = 1), colorectal cancer (*n* = 2), lung cancer (*n* = 5), and breast cancer cell lines (*n* = 14). After normalizing for variations in proliferation, there was a striking difference in the amount of cfDNA released across cancer cell lines (Fig. 1e). Interestingly, these trends were also observed when comparing the absolute quantities of DNA released from cells (Supplementary Fig. S1a). Examination of cfDNA fragmentation patterns across the cell line panel revealed two distinct fragmentation patterns. Cell lines demonstrated either “left-skewed” cfDNA with major peaks at around 167 bp reminiscent of patterns derived from human blood samples, or “right-skewed” cfDNA with largest peaks at sizes greater than 1000 bp (Fig. 1f, Supplementary Fig. S1b). Although media conditions varied across cell lines, differences in cfDNA release did not correlate with media differences and the switching of types of serum in culture media did not lead to large changes in fragmentation (Supplementary Fig. S1c). We also find that various serum types do not confound our experiments since they contain a dearth of DNA, as multiple of our cell lines were found to have almost no DNA present in their serum-enriched media (Fig. 1e, Supplementary Fig. S1a, b). The classification of each cell line into left- and right-skewed groups can be found in Supplementary Data 1, and overlaid fragmentation patterns of left- vs. right-skewed cell lines can be found in Supplementary Fig. 2a. Interestingly, right-skewed cell lines show overall greater cfDNA release capacity but do not show increased proliferation or cell death (Supplementary Fig. S2b, c, d, e). Furthermore, using the Cancer Cell Line Encyclopedia and Genentech databases^41,42^, the intrinsic expression of DNases across the cell lines was found to be generally very low and did not correlate with cfDNA release quantity or fragmentation pattern type (Supplementary Data 2, 3). Using RNA-seq from multiple TCGA cohorts, we confirmed that expression of DNases in human tumor tissue is similarly low (Supplementary Figs. S3a, b, c). Thus, local DNase expression in tumors does not account for the difference in fragmentation patterns between patient cfDNA and our in vitro data (Supplementary Fig. 3d, 3e, Supplementary Data 2, 3). Taken together, these results reveal a striking cell-intrinsic diversity of cfDNA release.

### cfCRISPR is a genome-wide cfDNA CRISPR-Cas9 screen that identifies putative modulators of cfDNA release

**Fig. 2:**
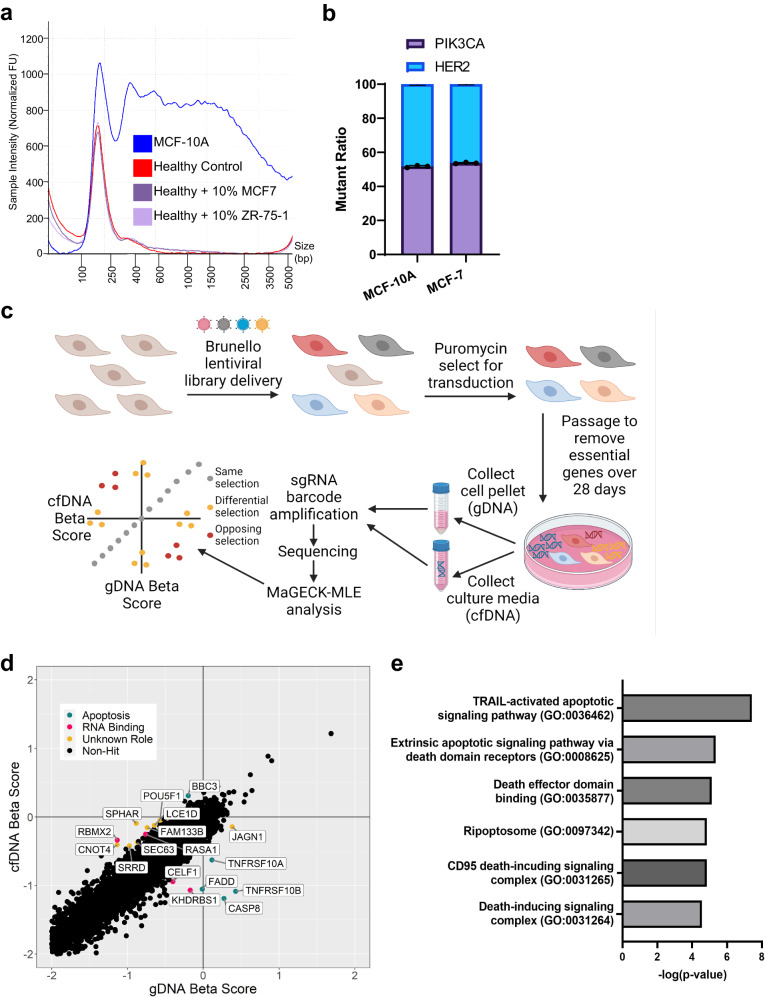
cfCRISPR is a genome-wide cfDNA CRISPR-Cas9 screen that identifies putative modulators of cfDNA release. **a** Fragmentation pattern of MCF-10A, healthy human donor control, and spike-in cfDNAs. Representative traces shown. **b** Relative representation of *PIK3CA* E545K and *ERBB2* L755S mutations in cfDNA from isogenically modified double mutant MCF-10As and MCF-7s. Ratio of mutant droplets was detected and quantified by ddPCR to determine relative representation of each locus. *n* = 3 biologically independent samples **c** Overview of the cell-free DNA CRISPR Screen methodology. **d** Genes plotted by their β-scores in both cfDNA and gDNA arms of MCF-10A CRISPR screen. Putative hits are highlighted and grouped by shared function (green, apoptosis; pink, RNA binding; yellow, unknown). **e** Gene ontology of genes from the MCF-10A CRIPSR screen determined as putative hits. Data are −log *p* values derived from PANTHER gene ontology and include enriched pathways in biological process, molecular function, and cellular component.

To identify regulators of cfDNA release, a new CRISPR screening strategy, cfCRISPR, was developed. MCF-10A is a nontumorigenic human breast epithelial cell line and was initially utilized for this screen due to its release of high amounts of relative and absolute cfDNA (Fig. 1e, Supplementary Fig. S1a). Additionally, MCF-10A cells display a left-skewed cfDNA fragmentation pattern reminiscent of that found in human plasma samples, including healthy controls, healthy controls with spiked-in cancer cell line cfDNA, and human cancer patients^43^ (Fig. 2a). We reasoned that if cfDNA is shed equivalently across a cell’s genome, then an integrated lentiviral sgRNA in that cell’s genome would be equally shed as cfDNA. If a gene knock-out affected the rate of cfDNA release, then the relative ratio of cfDNA to cellular genomic DNA (gDNA) for that particular sgRNA barcode would be skewed. To confirm cfDNA is shed equivalently across a cell’s genome, representative loci in MCF-10A and MCF-7 cfDNA were quantified using ddPCR. Equivalent representation of heterozygous mutations in *PIK3CA* and *ERBB2* previously knocked-in to these lines^44^ were detected for both cell lines (Fig. 2b), indicating that various genomic loci are likely evenly represented in cfDNA.

For the cfCRISPR screen, MCF-10A cells were infected with the Brunello Human CRISPR Knockout Pooled Library platform^45,46^. This library contains 76,441 sgRNAs targeting 19,114 genes. Infection led to a polyclonal population of cells that each contain 1 lentiviral particle and therefore one gene knockout. After puromycin selecting to remove cells that did not receive lentivirus, cells were passaged for 28 days prior to analysis to ensure the removal of essential genes to prevent false positives. At this timepoint, gDNA and cfDNA were isolated from polyclonal pool of cells adherent to the plate and the culture media, respectively. The relative representation of each gene’s barcodes was compared between the starting plasmid library and each endpoint DNA (gDNA or cfDNA) population using the MAGeCK-MLE algorithm^47^ (Fig. 2c). An output of this algorithm is the β-score, a representation of fold change between the starting plasmid library and either the endpoint gDNA or cfDNA arms of the screen. By identifying genes which were discordant in their β-score between the gDNA and cfDNA arms of the screen, we were able to identify genes which might regulate cfDNA release (Fig. 2d). For example, a gene whose knockout causes an increase in cell growth will be more represented in the gDNA population and therefore yield a high gDNA β-score. However, if that same gene’s cfDNA β-score is negative, the knockout causes an under-representation in the cfDNA pool. The discrepancy between the representation of this gene in the gDNA and cfDNA arms of the screen makes it a candidate modulator of cfDNA release. Genes which do not display a discrepancy between their cfDNA and gDNA β-score can be found along the Z-axis in Fig. 2d and are not likely candidate regulators of cfDNA release.

Putative genes of interest with β-scores discrepant between the cfDNA and gDNA arms of the screen were grouped based on literature-defined categories (Fig. 2d, Supplementary Data 4). Many of the candidates are well-known members of the TRAIL extrinsic-apoptotic cell-death pathway, including *TNFRSF10A*, *TNFRSF10B*, *CASP8*, and *FADD*. Multiple RNA binding, putative RNA binding, or proteins known to interact with these proteins were also identified, including *KHDRBS1*, *RBMX2*, and *CELF1*. PANTHER-based gene ontology^48^ revealed that based on biological process, molecular function, or cellular component, only pathways involved in extrinsic apoptotic pathways were enriched (Fig. 2e). These data suggest that pro-apoptotic genes in the TRAIL pathway, as well as other molecules with cryptic roles, regulate cfDNA release in MCF-10As.

### Sam68, FADD, and TRAIL modulate cfDNA release in human non-nontumorigenic MCF-10A cells through apoptotic pathways

**Fig. 3:**
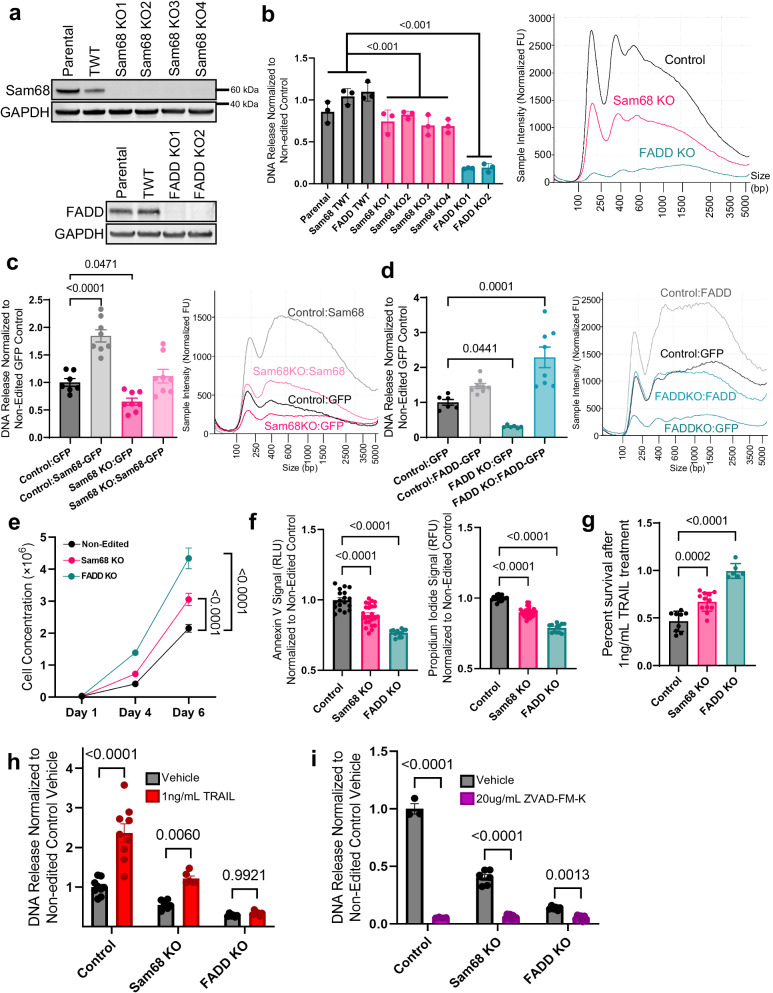
Sam68, FADD, and TRAIL modulate cfDNA release in human non-nontumorigenic MCF-10A cells through apoptotic pathways. **a** Immunoblot analysis of Sam68 and FADD after CRISPR-mediated knockout (KO) in the MCF-10A background. TWT = Targeted Wild-Type. **b** Quantification of DNA release from MCF-10A KO cell lines in culture. Individual cell lines shown, with data representing mean fold change ± SEM internally normalized to cell concentration for each cell line and then normalized to control; *n* = 3 biologically independent samples. Electropherograms were individually run at least *n* = 3 times and representative traces were selected. **c** Quantification of DNA release from Sam68 KO MCF-10A cell lines rescued by Sam68-GFP overexpression. Parental and TWT were grouped and labeled control, and two Sam68 KO3 and KO4 were grouped. Data represent mean fold change ± SEM internally normalized to cell concentration for each cell line and then normalized to control; *n* = 4 for all lines except *n* = 3 for Sam68 TWT before combining. Electropherograms were individually run at least *n* = 3 times and representative traces were selected. **d** Quantification of DNA release from FADD KO MCF-10A cell lines rescued by FADD-GFP overexpression. Parental and TWT were grouped and labeled control, and two FADD KO cell lines were grouped. Data represent mean fold change ± SEM internally normalized to cell concentration for each cell line and then normalized to control; *n* = 3 for all GFP-expressing lines and *n* = 4 for each FADD-GFP expressing line before combining. Electropherograms were individually run at least *n* = 3 times and representative traces were selected. **e** Cell growth assay of KO MCF-10A cell lines. Parental and TWT were grouped and labeled control. All four Sam68 KO cell lines and both FADD KO cell lines are respectively grouped. Data represent mean cell concentration ± SEM; *n* = 3 for each cell line before combining. **f** Annexin V and Propidium Iodide (PI) assay of KO MCF-10A cell lines. Parental and TWT were grouped and labeled control. All four Sam68 KO cell lines and both FADD KO cell were grouped, respectively. Data represent mean signal (RFU for PI; RLU for Annexin) ± SEM internally normalized to cell concentration for each cell line and then normalized to control; *n* = 6 biologically independent samples for each cell line before combining. **g** Cytotoxic assay of MCF-10A KO panel treated with 1 ng/mL TRAIL ligand. Parental and TWT were grouped and labeled control. All four Sam68 mutant cell lines and both FADD mutant cell lines were grouped, respectively. Data represent mean percent survival ± SEM as normalized to vehicle of each cell line condition; *n* = 3 biologically independent samples for each cell line before combining. **h** Quantification of DNA release from MCF-10A KO panel treated with 1 ng/mL TRAIL ligand. Parental and TWT were grouped and labeled control. All four Sam68 mutant cell lines and both FADD mutant cell lines were respectively grouped. Data represent mean fold change ± SEM internally normalized to cell concentration for each cell line and then normalized to control; *n* = 3 biologically independent samples for each cell line before combining. **i** Quantification of DNA release from MCF-10A KO cell lines treated with 20 μg/mL ZVAD-FM-K. Parental MCF-10As, Sam68 KO3/4 mutant cell lines, and both FADD mutant cell lines were respectively grouped. Data represent mean fold change ± SEM in DNA release internally normalized to cell concentration for each cell line and then normalized to control vehicle; *n* = 3 biologically independent samples for all untreated lines and FADD KO1, *n* = 4 for treated lines for each cell line before combining. All statistics were ANOVA with Dunnett’s multiple comparison test at endpoint.

To validate that the genes identified in our CRISPR screen were involved in cfDNA release, *KHDRBS1* (Sam68) and *FADD* (FADD) were selected for generation of CRISPR-mediated gene knockouts in MCF-10A cells. FADD is a central hub in the TRAIL pathway, as its recruitment by the trimerization of TRAIL receptors provides the scaffolding to activate caspase 8 cleavage and downstream apoptotic signaling^49^. Sam68 has a more cryptic role given its multifaceted functions in splicing, transcriptional/post-transcriptional gene regulation, and DNA damage response^50–52^, but was chosen as the top candidate among our putative RNA binding genes. Cell lines generated included four knockout cell lines using two distinct sgRNAs for *KHDRBS1* (Sam68 KO1/KO2 and KO3/KO4, respectively) and two knockout cell lines using one sgRNA for *FADD* (FADD KO1/KO2). Untreated cell lines (“parental”) and CRISPR-targeted single-cell clones that resulted in wild-type (“targeted wild-type”; TWT) were used as controls. Parental cells account for the natural phenotype of the cell line, while TWT cells control for the transfection process and off-target effects. Complete knockout in Sam68 KO and FADD KO lines was confirmed by sequencing and immunoblot (Fig. 3a, Supplementary Fig. S4a, b, c). Our CRISPR screen analysis (Fig. 2d) predicts the directional effect of knockout on cfDNA release—hits below the Z-axis will likely lead to decreases in cfDNA release when knocked out, whereas those above the Z-axis will likely lead to increases. Sam68/*KHDRBS1* and *FADD* are positioned below this Z-axis. Their low position on the y-axis represents negative selection in the cfDNA arm of our screen while its near zero β-score in the gDNA arm represented on the x-axis indicates a lack of selection. Therefore, we would expect that knockout of these genes would lead to decreased cfDNA release as they were not selected against cellularly, but less of their DNA was found in culture media than expected. Indeed, analysis of cfDNA release from our knockout cell lines confirmed that knockout of Sam68 or FADD lead to a significant decrease in cfDNA release as expected, with FADD KOs demonstrating a greater than 75% decrease (Fig. 3b). Analyses using multiple KO cell lines are collapsed in future figures by genotype, with Supplementary Fig. S5 representing the combined version of this analysis. DNA fragmentation analysis of these samples displayed a decrease in cfDNA release across all fragment sizes (Fig. 3b). Re-expression of GFP-tagged Sam68 and FADD proteins in their respective knockout cell lines was able to fully rescue cfDNA release, while maintaining the cell line’s innate left-skewed fragmentation pattern. Overexpression of each protein in the parental or TWT MCF-10A control cell lines concordantly led to a significant increase in cfDNA release. Though this cell line is left-skewed at baseline, overexpression of both proteins led to a dominant peak at ~400 bp (Fig. 3c, d). Further characterization of the knockout cell lines revealed an increased rate of growth when compared to the non-edited controls (Fig. 3e). To determine whether this was due to increased cell proliferation or decreased cell death, cells were analyzed for cell death markers. Interestingly, all knockout lines demonstrated decreased early apoptotic cell death initiation through Annexin V labeling and decreased membrane permeability with propidium iodide (PI) compared to the control lines (Fig. 3f). These changes indicate that differential cell growth is due, at least in part, to alterations in apoptotic pathways. Given the top cfCRISPR screen candidates were primarily in the TRAIL pathway, the knockouts were next evaluated to determine if they were resistant to TRAIL-induced apoptotic cell death. Both Sam68 and FADD knockout lines were resistant to TRAIL-induced apoptosis, with FADD knockouts demonstrating complete resistance, and Sam68 showing partial resistance (Fig. 3g). In turn, TRAIL administration led to increased cfDNA release in the non-edited controls as well as a muted increase in the partially resistant Sam68 knockout lines but did not alter release in FADD knockouts (Fig. 3h). We also performed a similar analysis in the MDA-MB-468 breast cancer cell line, finding that Sam68 or FADD knockout alone did not lead to changes in cfDNA release. However, upon TRAIL administration Sam68 and FADD knockout lines displayed decreased cfDNA release compared to treated wild-type cells, further validating Sam68 and FADD as mediators of the TRAIL pathway and cfDNA release (Supplementary Figs. S6a, b). Furthermore, treatment with the pan-caspase inhibitor ZVAD-FM-K abrogated cfDNA release in all MCF-10A derivative cell lines (Fig. 3i). Although this inhibitor can prevent lysosome-dependent and pyroptotic cell death in addition to apoptotic cell death, to our knowledge, MCF-10A cells grown in culture would not be susceptible to these alternative forms of cell death without additional stimuli. These data taken together indicate that caspase-dependent apoptotic cell death is a major source of cfDNA release in vitro.

The role of FADD as a mediator of the extrinsic apoptosis pathway is well-defined in the literature, but the role of Sam68 in cell death is less understood. A previous group demonstrated Sam68 interacts with the TNFα receptor complex to promote apoptosis and NF-κB activation^53^, suggesting Sam68 might also have a direct role in signaling at the TNFR superfamily member TRAIL receptor^54–57^. To determine the potential pathway overlap of Sam68 and FADD, we performed a double knockout of FADD and Sam68 in the MCF-10A background (Supplementary Fig. S7a). We saw that double knockout cells did not display further decreased cfDNA release compared to FADD only mutants, indicating that these two genes likely are involved in the same pathway (Supplementary Fig. S7b). To further define the interplay between FADD and Sam68, we overexpressed FADD and Sam68 in opposing knockout cell lines. In doing so, we found that FADD overexpression in a Sam68 knockout led to an increase in cfDNA release, while Sam68 overexpression in a FADD knockout background did not lead to rescue (Supplementary Fig. S7c). This implies that FADD is required for Sam68’s impact on cfDNA release, but Sam68 is not required for FADD’s function. In terms of evidence for a direct physical interaction at the TRAIL receptor, previous literature suggests that Sam68 can be both a nuclear protein and a cytoplasmic protein^50,58,59^. Within our models, nuclear/cytoplasmic fractionation of endogenous Sam68 and the localization of an overexpressed Sam68-GFP fusion protein revealed Sam68 is exclusively a nuclear protein and is likely not involved in receptor activation with FADD in our models (Supplementary Figs. S8a, b). These results indicate that Sam68 plays a role in apoptotic regulation and FADD is required for this function, but the mechanistic link between these two genes in our model system remains unknown. Previous studies have shown that Sam68 can splice the BCL-X mRNA product of the *BCL2L1* gene, and depletion of Sam68 leads to accumulation of the anti-apoptotic product BCL-XL spliceoform over the pro apoptotic BCL-XS^60,61^. Given that Sam68 knockout MCF-10A cells display decreased apoptosis, we hypothesized that Sam68 may be mediating the splicing of this apoptosis-related protein. However, we were unable to observe differences in the expression of these products at the mRNA level by RT-PCR (Supplementary Fig. S8c). Further delineation of Sam68’s role in apoptosis and the TRAIL pathway will be the focus of future study.

### Sam68, FADD, and TRAIL modulate cfDNA release across cancer cell lines through apoptotic pathways

**Fig. 4:**
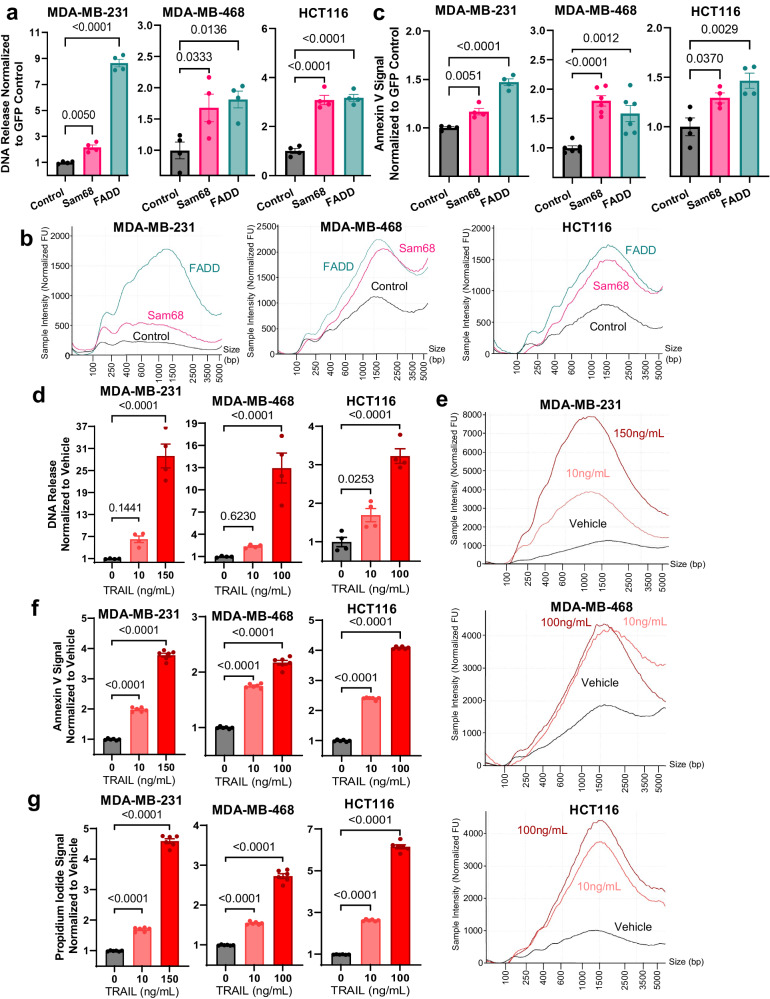
Sam68, FADD, and TRAIL modulate cfDNA release across cancer cell lines through apoptotic pathways. **a** Quantification of DNA release from MDA-MB-231, MDA-MB-468, and HCT116 cancer cell lines with overexpression of GFP-tagged Sam68 or FADD. Data represent mean fold change ± SEM in DNA release normalized to cell concentration for each cell line, then overall to GFP control; *n* = 4 biologically independent samples. **b** Fragmentation pattern of MDA-MB-231, MDA-MB-468, and HCT116 cancer cell lines with overexpression of GFP-tagged Sam68 and FADD. Electropherograms were individually run at least *n* = 3 times and representative traces were selected. **c** Quantification of Annexin V signal from MDA-MB-231, MDA-MB-468, and HCT116 cancer cell lines with overexpression of GFP-tagged Sam68 and FADD. Data represent mean fold change ± SEM in RLU signal normalized to cell concentration at collection for each cell line, then overall to GFP control; *n* = 4 biologically independent samples for MDA-MB-231 and HCT116, *n* = 6 for MDA-MB-468. **d** Quantification of DNA release from MDA-MB-231, MDA-MB-468, and HCT116 cancer cell lines treated with TRAIL ligand. Data represent mean fold change ± SEM in DNA release normalized to cell concentration for each treatment, then overall to vehicle control; *n* = 4 biologically independent samples. **e** Fragmentation pattern of MDA-MB-231, MDA-MB-468, and HCT116 cancer cell lines treated with TRAIL ligand. Electropherograms were individually run at least *n* = 3 times and representative traces were selected. **f** Quantification of Annexin V signal from MDA-MB-231, MDA-MB-468, and HCT116 cancer cell lines treated with TRAIL Ligand. Data represent mean fold change ± SEM RLU signal normalized to cell concentration for each treatment, then overall to vehicle control; *n* = 6 biologically independent samples. **g** Quantification of Propidium Iodide signal from MDA-MB-231, MDA-MB-468, and HCT116 cancer cell lines treated with TRAIL Ligand. Data represent mean fold change ± SEM RFU signal normalized to cell concentration for each treatment, then overall to vehicle control; *n* = 6 biologically independent samples. All statistics were ANOVA with Dunnett’s multiple comparison test at endpoint.

To determine if the screen-identified mediators of apoptosis affect cfDNA release in human cancer cells, GFP-tagged Sam68 and FADD were overexpressed in five different cancer cell lines. Overexpression increased cfDNA release across all lines, with the most pronounced increases in the FADD overexpressing cell lines (Fig. 4a, Supplementary Fig. S9a). These increases in cfDNA release led to an increase in cfDNA released at large fragmentation sizes, resulting in a right-skew in all cell lines (Fig. 4b, Supplementary Fig. S9b). Overexpression of Sam68 or FADD resulted in significantly increased Annexin V signal in most lines, indicating increased propensity for apoptosis (Fig. 4c, Supplementary Fig. S9c). Membrane permeability via PI was not measured due to fluorescence overlap with GFP. The same cancer cell lines were then exposed to TRAIL ligand, and a dose dependent increase in cfDNA release was observed for all cell lines (Fig. 4d, Supplementary Fig. S9d). Again, this manipulation increased cfDNA release primarily at larger fragment sizes (Fig. 4e, Supplementary Fig. S9e). These gains in cfDNA release were associated with increases in both Annexin V and PI assays, suggesting these responses were mediated by increased apoptosis (Fig. 4f, g, Supplementary Fig. S9f, g).

### Generalization of apoptosis as a mediator of cfDNA release in cancer cells

**Fig. 5:**
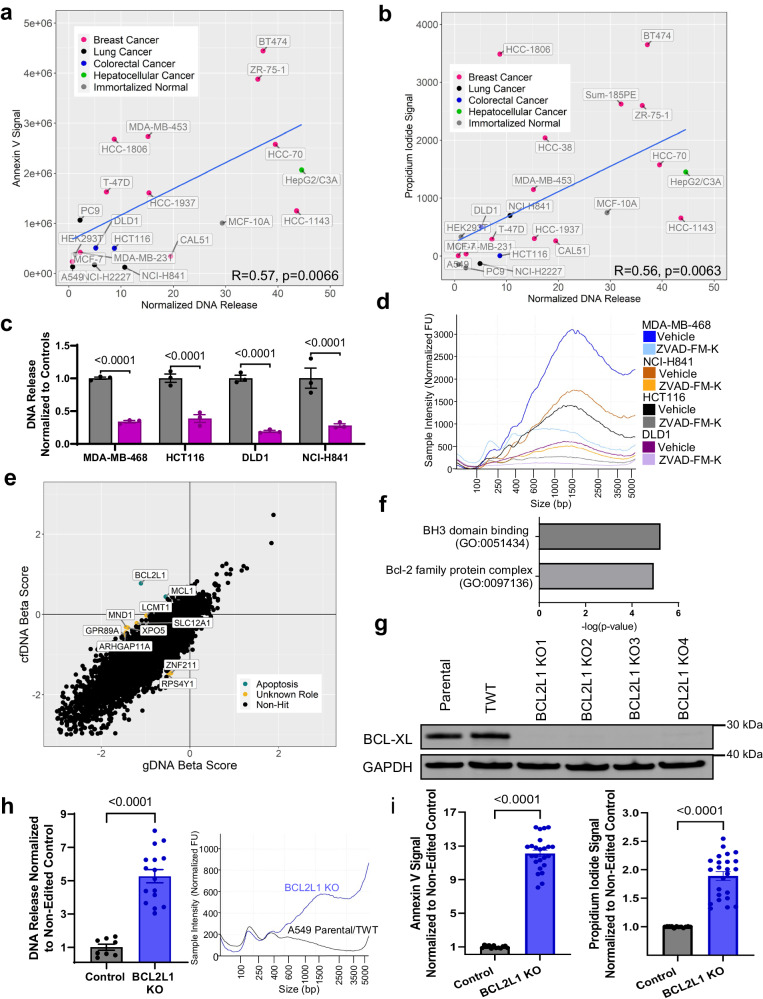
Apoptosis is the major controller of cfDNA release across nontumorigenic and cancer cell lines. Correlation of Annexin V (**a**) and Propidium Iodide (**b**) signal to cfDNA release. Data represent mean DNA release normalized to cell concentration and Annexin V or PI signal (RLU or RLU, respectively) normalized to cell concentration; *n* = 3 for each cell line, statistics were Pearson correlation with outlier removal using the ROUT method. Out of the 24 cell lines in the panel, 3 were removed in (**a**) and 2 were removed in (**b**). **c** Quantification of DNA release from cancer cell lines upon caspase inhibition with 20 μM ZVAD-FM-K. Data represent mean fold change ± SEM in DNA release normalized to cell concentration for each cell line, then to vehicle; *n* = 3 biologically independent samples. **d** Electropherograms were individually run at least *n* = 3 times and representative traces were selected for samples quantified in (**c**). **e** Genes plotted by their β-scores in both cfDNA and gDNA arms of A549 CRISPR screen. Putative hits are highlighted and grouped by shared function (green, apoptotic; yellow, unknown). **f** Gene ontology of genes from the MCF-10A screen determined as putative hits. Data are -log p-values derived from PANTHER gene ontology, and include enriched pathways in biological process, molecular function, and cellular component. **g** Immunoblot analysis of BCL-XL after CRISPR-mediated knockout (KO) in the A549 background. TWT = Targeted Wild-Type. **h** Quantification of DNA release from A549 KO cell lines in culture. Parental cells and TWT cells were averaged and labeled control. All four *BCL2L1* KO cell lines were grouped. Data represent mean fold change ± SEM in DNA release normalized to cell concentration for each cell line then normalized to control; *n* = 4 biologically independent samples for each cell line before combining. Electropherograms were individually run at least *n* = 3 times and representative traces were selected. **i** Annexin V and Propidium Iodide (PI) assay on all generated A549 cell lines. Parental cells and TWT cells are combined. All four *BCL2L1* KO cell lines were grouped. Data represent mean signal (RFU for PI or RLU for Annexin) ± SEM normalized to cell concentration then overall to Control; *n* = 6 biologically independent samples for each cell line before combining. All statistical significance shown was derived from student’s *t* test.

To further elucidate the relationship between cfDNA release and apoptosis, Annexin V and PI signals were next quantified across the 24-cell line panel and compared with cfDNA release. There was a significant correlation between cfDNA release and both baseline apoptotic indices (Fig. 5a, b). In addition, treatment with the pan-caspase inhibitor ZVAD-FM-K decreased cfDNA release across all cell lines but did not alter overall rightward vs. leftward fragmentation pattern skewing, suggesting again that apoptotic DNA release does not consistently lead to the putative apoptotic fragmentation pattern of 167 bp with a continuing ladder pattern (Fig. 5c, d).

To unequivocally verify apoptotic pathways as a mediator of cfDNA release, we performed a second genome-wide CRISPR screen using the low cfDNA-releasing human lung cancer cell line, A549 (Fig. 5e) to identify gene knockouts that would increase cfDNA release. *BCL2L1* and *MCL1* were identified as significant candidates and both are members of the BCL-2 family (Supplementary Data 5). No other significant gene families were identified by gene ontology (Fig. 5f). BCL-XL, one of the gene products of *BCL2L1*, as well as MCL-1, are both anti-apoptotic multi-domain members of the BCL-2 family that regulate apoptosis by preventing mitochondrial membrane permeabilization^62^. To test whether these genes negatively regulated cfDNA release, four *BCL2L1* knockout cell lines were generated using two sgRNAs (Fig. 5g, Supplementary Fig. S10a, S10b). Interestingly, cfDNA release increased but the fragmentation pattern of the knockout lines was skewed towards larger fragments (Fig. 5h) in this typically left-skewing cell line. This change was concomitant with increases in baseline Annexin V and PI signal (Fig. 5i), indicating that these effects were likely due to apoptosis. Knockout of *BCL2L1* was also performed in two cell lines without single cell dilution (Supplementary Fig. S11a). This CRISPR “knockdown” in a bulk population led to an increase in cfDNA release in two cell lines tested (Supplementary Fig. S11b) akin to the *BCL2L1* knock out in A549 cells.

Taken together, our results confirm that the apoptotic pathway is a major driver of cfDNA release from human normal and cancer cells. Our findings also challenge the assumption that DNA released by apoptotic cells into the bloodstream is fully processed to a modal size of 167 bp, and alternatively we propose this fragmentation is due to additional DNA degradation in the bloodstream after cfDNA is shed by primarily apoptotic processes as large fragments. This hypothesis of further degradation in the bloodstream of patients is supported by low DNAse expression in local tumor tissues (Supplementary Fig. S3), indicating circulating DNAses likely perform this function. Importantly, we demonstrate that cfDNA release can be modified across various cell lines using a variety of apoptotic manipulations, providing opportunities for diagnostic improvements as discussed below.

## Discussion

cfDNA is now routinely used in the clinical setting to help guide the diagnosis and treatment of patients in many distinct areas of medicine. In particular, for oncology ctDNA-based liquid biopsies have shown great promise as tools for guiding cancer treatment but continue to have limitations in sensitivity and negative predictive value^63^. Most attempts to address these shortcomings have evaluated the use of additional analytes or applied advanced sequencing bioinformatic techniques, although a recent study has also implicated pre-treatment with inhibitors of DNA degradation^11–14,64–67^. However, the mechanisms of how cfDNA is liberated into circulation has not been proposed as a potential means of increasing cfDNA release and therefore sensitivity of liquid biopsies. Herein, we present genetic evidence that apoptotic pathways are a major regulator of cfDNA release using cfDNA-based CRISPR screens, single gene knockouts, overexpression rescue studies, and drug treatments across multiple human cell lines. In contrast to previous literature suggesting roles for vesicles as well as necrosis in cfDNA/ctDNA release^32,34–37,68^, our screens did not implicate these processes. Our DNA isolation methods purposefully retain most large and small vesicle populations, and the lack of vesicle-related genes seen in both screens indicates a minor or null role for vesicle populations on cfDNA release. Importantly, we instead show that cfDNA released from human cells is primarily derived from apoptotic pathways. Even Sam68, a known RNA binding protein, mediated its effects on cfDNA release in part through apoptotic pathways as measured by changes in Annexin V and other biologic parameters. Interestingly, our screens in the MCF-10A and A549 cell lines revealed hits in different parts of the apoptotic pathway, with *FADD* in the MCF-10A screen being a mediator of extrinsic cell death pathways and *BCL2L1* in the A549 screen being an inhibitor of intrinsic apoptosis. The identification of these cell line-specific hits reflects the biological differences between the two cell lines. A549 cells are known to be resistant to TRAIL ligand, and MCF-10As display very low expression of BCL-XL, the apoptosis-inhibiting spliceoform of *BCL2L1*^69–71^. Thus, it would have been surprising to see these hits or other similar ones identified across both screens. The convergence of non-overlapping hits from both screens onto the same overarching biological process of apoptosis confirms the validity of our findings and is not uncommon in studies CRISPR screening in multiple cell types^72,73^. Taken together, these screens support our findings that apoptosis is a major mediator of cfDNA release. However, hits we generated but did not explore (Supplementary Data 4, 5) could represent additional pathways that regulate cfDNA release. Though we did not explore many of these hits, due in part to the relative obscurity of many of the genes identified, they may represent exciting targets for future study.

We also found that the fragmentation pattern of DNA released through these apoptotic pathways can contain larger sizes traditionally assumed to be released through vesicular or necrotic pathways, rather than the expected 167 bp size expected following apoptotic DNA cleavage. This was visualized in multiple right-skewed cell lines increasing cfDNA quantities at sizes >1000 bp after apoptosis induction through Sam68, FADD, and TRAIL treatment. In addition, the left-skewed A549 began skewing rightwards upon loss of anti-apoptotic *BCL2L1*, and the left-skewed MCF-10A began skewing more rightwards upon overexpression of pro-apoptotic Sam68 and FADD. This suggests that apoptotic DNA is not always released from cells at the smaller “apoptotic” sizes seen in patient blood samples and assumed in the literature, but rather may undergo post-release processing in the blood that results in smaller “left-skewed” sizes akin to those found in our cell-free DNA degradation experiments.

In addition, we present a new cfDNA CRISPR screening modality in cfCRISPR that may prove useful for other applications. Traditionally, CRISPR screens require the isolation of DNA from tens of millions of cells at any given timepoint. In analyzing cfDNA, we saw that most genes were not discrepant between genomic and cell-free DNA in their representation. This provides possibilities for future screens to serially identify genes of interest over time using sequential harvesting of culture media containing cfDNA instead of or in addition to gDNA. In theory this also opens the door to use CRISPR screens to study other DNA-related processes, such as DNA trafficking into multi-vesicular bodies by harvesting DNA from non-nuclear populations^40^. Thus, in this work we not only improve our understanding of cfDNA biology, but also provide a new platform to perform genome-wide CRISPR screens in non-nuclear DNA populations.

While our results come from in vitro models, we believe they may have implications for in vivo and patient settings, to be assessed in future studies. The finding that we can increase or decrease cfDNA and ctDNA by specific gene knock out in vitro raises provocative questions with clinical implications. For example, if a cancer patient has a high variant allele fraction for a specific mutation with ctDNA testing, does this necessarily represent the predominant clonal population or a subclone that is prone to higher cfDNA release due to inactivation or activation of certain genes? In addition, although recombinant TRAIL and agonist TRAIL receptor therapies did not demonstrate efficacy in clinical trials, there is the possibility that these drugs, as well as many other agents with distinct functions, could be repurposed as “ctDNA adjuvant diagnostics”. Although such drugs may increase both ctDNA and total cfDNA release from normal cells, this may still provide meaningful benefit since the sensitivity and negative predictive value of liquid biopsies are often limited by the lack of total cfDNA, and not necessarily by the relatively low amount of ctDNA to total cfDNA. Indeed, some current commercial ctDNA assays state a technical lower limit of detection well below one mutant molecule per 100,000 wildtype cfDNA molecules (0.001% allele fraction)^74^. However, 100,000 cfDNA molecules is equivalent to ~300 ng of cfDNA (one haploid genome = 3 picograms of DNA), and the amount of cfDNA obtained for clinical testing is often far below this threshold to achieve such sensitivity and therefore false negatives remain problematic. Increasing total cfDNA, along with ctDNA, could solve this current unmet need, and allow for highly sensitivity and specific testing that may guide clinical care with precision.

## Materials and methods

### Cell lines

A complete list of cell lines used can be found in Supplementary Data 6. MCF-10A, MCF-7, T-47D, BT474, ZR-75-1, MDA-MB-231, HCT116, and DLD1 cells were acquired from the American Type Culture Collection (ATCC). HepG2/C3A and HEK293T cells were kindly provided by Dr. Emily Hodges (Vanderbilt University). CAL51, Sum-185PE, MDA-MB-453, MDA-MB-468, HCC38, HCC70, HCC1143, HCC1937, and HCC1806 were provided by Dr. Brian Lehmann (Vanderbilt University Medical Center). A549, PC9, NCI-H841, NCI-H1607, and NCI-H2227 were provided by Dr. Christine Lovly (Vanderbilt University Medical Center). MCF-10A cells were grown in DMEM:F12 (1:1) (GIBCO) supplemented with 5% horse serum (Life Technologies), 20 ng/mL epidermal growth factor (EGF; Sigma-Aldrich), 10 μg/mL insulin (Life Technologies), 0.5 μg/mL hydrocortisone (Sigma-Aldrich), 0.1 μg/mL cholera toxin (Sigma-Aldrich), and 1% penicillin-streptomycin (PS; Life Technologies). Sum185-PE cells were grown in MCF-10A media replacing horse serum with fetal bovine serum (FBS; Life Technologies), doubling the concentration of hydrocortisone and halving that of insulin. MCF-7, BT474, ZR-75-1, CAL51, HepG2/C3A, HEK293T, HCT116, MDA-MB-231, MDA-MB-453, and MDA-MB-468 were grown in DMEM supplemented with 10% FBS and 1% PS. A549, T-47D, DLD1, PC9, HCC38, HCC70, HCC1143, HCC1806, HCC1937, NCI-H841, NCI-H1607, and NCI-H2227 were all grown in RPMI supplemented with 10% FBS and 1% PS. The cell lines used were verified by STR profiling.

### cfDNA release assays

Cell lines and their derivatives were plated in T75 plates at the following densities: 4 × 10^5^ for MCF-10A, 7.5 × 10^5^ for A549, HCT116, DLD1, MDA-MB-231, NCI-H841, and 1 × 10^6^ for MDA-MB-468. 24 h after seeding, the media was replaced to a volume of 10 mL in T75. After media change, cells were allowed to grow to ~85% confluency over the next 3 days. Upon reaching this density, media was collected and centrifuged to remove live cells, then dead cells and debris at 300 × *g* for 10 min and 2000 × *g* for 30 min, respectively. DNA was isolated from 8 mLs of media from T75s through the QIAamp MinElute ccfDNA Midi Kit (Qiagen), used according to manufacturer protocols. All DNA was eluted in 25 µL of provided deionized water. This approach deliberately leaves most large and small vesicle subtypes in the isolated media and therefore would include any DNA they might carry. Concentrations of DNA were measured by fluorescence using the Quant-iT dsDNA Assay Kit High Sensitivity or Quant-iT dsDNA Assay Kit Broad Range (Invitrogen) on the GloMax Discover system (Promega). Simultaneously, cell counts present on the plate were measured by Vi-Cell BLU Cell Viability Analyzer (Beckmann-Coulter). DNA concentrations were divided by total cell concentrations to normalize the data for cell growth differences. In studies where there was a vehicle, control, or non-edited cell line, the data was normalized to these groups. Samples were analyzed for fragmentation analysis using D5000 ScreenTape on TapeStation 2200 or 4200 (Agilent) according to the manufacturer’s specifications. All assays were performed in at least triplicate. All drug assays used the indicated concentration of TRAIL ligand (Millipore Sigma, GF092) or Z-VAD-FMK (Selleck Chemicals, S7203).

### cfDNA panel studies

Cells were all plated at 4 × 10^5^ cells per plate in three T75 cell culture flasks. The following day media was changed to remove nonadherent cells and debris. Three days later, media from all cell lines was collected, isolated, and quantified as described in the cfDNA release assays section. Fragmentation patterns were taken on an Agilent Tapestation 2200 or 4200. Representative fragmentation patterns were taken from initial panel samples or were products of assays optimized for higher seeding densities to maximize DNA release and detection. Average values of normalized and non-normalized cfDNA release were correlated with various parameters, including Annexin V/PI values derived for each cell line as described below in the “Baseline cell death assay section” and DNAse expression levels derived from Cancer Cell Line Encyclopedia and Genentech databases.

### Cell growth assay

Exponentially growing MCF-10A cells of each knockout genotype were plated at 2 × 10^3^ cells per well in six-well plates. On indicated days, cells were counted using a Beckman Coulter Vi-Cell BLU Cell Viability Analyzer. All cell lines were counted in triplicate.

### Released DNA degradation assay

Cells were plated and assayed as described in the cfDNA Release Assays section, with the initial media replaced to a volume of 13 mL. After incubation for 3 days, media was isolated as described above. 4 mL of media was taken for Day 0 and frozen down at −20 °C. The remaining 8 mL were placed back in the cell culture incubator in a 15 mL Falcon tube and additional 4 mL collections were taken on days 3 and 7. Media was thawed simultaneously and isolated and quantified as described above.

### DNA release time-course assays

Cells were plated and assayed as described in the cfDNA Release Assays section. Analysis was performed on at least three replicates on days 1, 2, and 3. DNA was isolated from media and quantified as described in the cfDNA Release Assay section.

### CRISPR screening

The Brunello Human CRISPR Knockout Pooled Library was purchased from Addgene as a lentivirus (#73179-LV). This library generally employs four guides per gene in the human genome, as well as 1000 non-targeting guides. The workflow for these screens is delineated in Fig. 2c. One biological replicate was performed for each screen. First, a titering assay was performed with a small aliquot of the virus for each cell line using either reverse infection (MCF-10A) or spinfection (A549) as outlined in the Broad Genome Perturbation Web Portal Protocols (https://portals.broadinstitute.org/gpp/public/resources/protocols). In short, cell lines were seeded in antibiotic-free media at 100,000 cells per well in a 6-well plate for reverse infection or 3 million cells per well in a 12 well plate for spinfection. Virus was added in varying quantities to each well and either left for 18 h for infection in tissue culture incubators or spun at 931 g for 2 h at 30 degrees Celsius prior to 18 h incubation. Media was then replaced with complete media, and 48 h after infection the various conditions were harvested and replica-plated to compare between puromycin-selected and non-selected conditions. The quantity of virus where 30–50% of cells survived selection was chosen and used for the full screen. Cell lines were infected at this concentration to achieve an MOI of 0.3–0.5 at a guide depth of 400X. Transduced cells were selected with puromycin (Thermo Fisher Scientific) for 3–5 days at 1 μg/mL (MCF-10A) or 2 μg/mL (A549). After selection, cells were maintained in culture for 28 days to eliminate any essential genes which might contaminate the pool of cfDNAs. At 25 days, cells were seeded to reach 90% confluency on day 3 and cfDNA and gDNA were extracted, respectively. At the end of each screen, at least 3 × 10^7^ cells were collected to maintain 400X guide depth, and all media from each plate was collected. cfDNA was extracted from the media using the Quick-DNA Urine Kit (Zymo) and combined. gDNA was extracted using the QiaAMP DNA Blood Maxi Kit (Qiagen). The sgRNA sequences were amplified and sequencing adapters were added, following the protocol outlined in the Broad Genome Perturbation Platform, using Phusion High-Fidelity PCR Master Mix (Thermo Scientific). Amplified samples were submitted and sent to the VANTAGE genomics core at Vanderbilt University Medical Center for sequencing. Analysis of read counts, β-score calculation, and *p* values was performed through the MAGeCK-MLE algorithm, comparing the initial plasmid pool to the gDNA and cfDNA arms. This algorithm initially compares the presence of each gRNA barcode individually, then collapses guides against the same gene when providing β-scores, a measure of fold change. Genes were considered putative hits when the absolute value of the β-score difference between the cfDNA and gDNA portions of the screen were >0.5 for MCF-10A or >0.95 for A549 and when the gene was significantly selected (*P* < 0.05 and FDR < 0.1) in one arm of the screen but not the other or in different directions in each arm of the screen.

### CRISPR gene knockout and cell-line generation

CRISPR gene knockout was performed by ribonuclear protein (RNP) transfection in the method recommended by Addgene, (https://www.idtdna.com/pages/support/guides-and-protocols), as described below. Single-guide RNAs (sgRNA) were identified from initial Brunello CRISPR Knockout Pooled Library and commercially synthesized (IDT). Specific guides and primers used to sequence the regions where the guides cut can be found in Supplementary Data 6. The RNP complex was assembled by incubating 1 µM Alt-R CRISPR-Cas9 sgRNA (IDT), 1 µM Alt-R S.P. HiFi Cas9 Nuclease V3 (IDT), and Cas9 PLUS Reagent (Invitrogen) with Opti-MEM (GIBCO) for 5 min at room temperature. Transfection complexes were formed by incubating assembled RNPs with CRISPRMAX transfection reagent in Opti-MEM for 20 min. Transfection complexes were plated first into 96-well plates followed by addition of cells such that the final concentration of cells/well was 40,000 and the final concentration of RNP was 10 nM. Cells were incubated with transfection complexes in a tissue culture incubator for 48 h and were subsequently single-cell diluted to create clonal populations. Selected clones were confirmed for targeted knockout by Sanger sequencing and immunoblot. Sanger sequencing was performed through Azenta Life Sciences. For CRISPR cell pools, single-cell dilution was not performed and cells were allowed to grow to confluency, at which point protein was harvested and the cells were seeded for assay.

### Immunoblot analysis

Cells were seeded in respective normal growth media and harvested during passages for protein lysates. Cells were lysed in RIPA Lysis and Extraction Buffer (ThermoFisher, 89900) supplemented with complete EDTA-free Protease Inhibitor Cocktail (Millipore Sigma, 04693159001) and PhosSTOP Phosphatase Inhibitor Cocktail (Millipore Sigma, PHOSS-RO) Tablets. Lysates were sonicated and protein concentrations were measured using the Microplate BCA Protein Assay Kit (Thermo Scientific, 23252). Samples were diluted and normalized in 4X NuPAGE LDS Sample Buffer (Invitrogen, NP0007) with 5% beta-mercaptoethanol (Aldrich) and were heated for 10 min at 70 °C. Protein lysates were then resolved by SDS-PAGE using NuPAGE 4–12% Bis-Tris 1.0–1.5 mm Mini Protein Gels and transferred onto PVDF membranes (Invitrogen, IB24002). After a 2-h incubation at room temperature with 5% BSA in TBST blocking buffer, blots were incubated overnight at 4 °C in blocking buffer with primary antibody. Blots were washed three times in TBST before incubation with fluorescent or chemiluminescent secondary antibodies. Images were taken on the ChemiDoc MP Imaging System (BioRad). Cell fractionation was performed using the NE-PER Nuclear and Cytoplasmic Extraction Reagents in place of the above technique for cellular fractionation experiments (ThermoFisher, 78833). Antibodies used in these studies can be found in Supplementary Data 6 and are as follows: Lamin A/C (1:1000, Cell Signaling Technologies, 2032), α-tubulin (1:1000, Abcam, ab4074), Sam68 (1:500, Santa Cruz, sc-1238), FADD (1:1000, Abcam, ab108601), GAPDH (1:1000, Cell Signaling Technologies, 5174), BCL-XL (1:1000, Cell Signaling Technologies, 2764), Goat anti-Rabbit IgG (H + L) Alexa Fluor Plus 647 (1:10,000 ThermoFisher, A32733), Goat anti-Rabbit IgG (H + L) Alexa Fluor Plus 488 (1:10,000, ThermoFisher, A32731), Goat anti-Mouse IgG (H + L) Alexa Fluor 488 (1:10,000, ThermoFisher, A11029), Digital anti-Mouse-HRP (1:1000, KwikQuant, R1005), and Digital anti-Rabbit-HRP (1:1000, KwikQuant, R1006).

### Baseline cell death assay

Cells were plated at 2 × 10^4^ cells per well in clear-bottomed white 96 well plates (Greiner-Bio One). The next day, the media was replaced with 100 μL growth media, and 100 μL 2X Detection Reagent prepared from the RealTime-Glo Annexin V Apoptosis and Necrosis Assay Kit (Promega). Plates were incubated for 24 h and read for fluorescence and luminescence as directed by Promega on the Glomax Discovery system. Cells were then trypsinized and counted within each well using the Vi-Cell BLU Cell Viability Analyzer, and signal was normalized to the average concentration of cells in wells for each cell type. In studies where there was a vehicle, control, or non-edited cell line, the data was normalized to these groups. When used to profile the cell line panel, background resultant from the usage of different media types was subtracted prior to normalization to cell counts.

### Stable overexpression and re-expression cell line generation

Lentiviral expression vectors with CMV promoters driving GFP-tagged human Sam68 and FADD were purchased from Origene (PS100093, RC200263L4, RC201805L4). Lentiviral particles containing these vectors were isolated using the Lenti-vpak Lentiviral Packaging Kit (Origene, TR30037) as directed by the manufacturer. When ready to transduce, lentivirus was thawed rapidly at 37 °C. Cells were seeded 50,000 cells in 1 mL maintenance media without any antibiotics into 6 well plates and reverse transduced with 500 μL of virus per well. Control wells were seeded in the absence of virus. After 48 h, cell lines were selected with puromycin at the following doses: MCF-10A .4 μg/mL for selection and maintenance, all cells grown in DMEM were selected at 2 μg/mL and maintained at 0.5 μg/mL, and all cells grown in RPMI were selected at 0.5 μg/mL and maintained at 0.25 μg/mL. After selection, cells were then flow-sorted at the Vanderbilt Flow Cytometry Shared Resource on the FACSAria III (BD) for the top 1% of GFP expression from the baseline cell pools and utilized in indicated experiments.

### RT-PCR splicing assay

Primers were designed that would simultaneously amplify BCL-XL and BCL-XS splice products of *BCL2L1*. Primer sequences can be found in Supplementary Data 6. Control cells and Sam68 KO cells were plated at 300,000 cells per well in 6 well plates using standard MCF-10As growth media as described previously. Cells were allowed to grow to 80% confluence over 2 days. Cells were then harvested for RNA using the RNAeasy Mini Kit (Qiagen, 74104). Equivalent 1 μg quantities of RNA were added into iScript cDNA Synthesis Kit (BioRad, 1708890) and converted to cDNA by manufacturers’ protocols. PCRs were performed with primers described above using Phusion Hot Start II High-Fidelity Master Mix (ThermoFisher, F565L) with an annealing temperature of 63 degrees Celsius. Samples were then run on an agarose gel and visualized by UV light with GelRed (Biotium, 41003).

### Digital droplet PCR

Custom primers and probes were developed and used as indicated against the *PIK3CA* E545K, and *ERBB2* L755S mutations and can be found in Supplementary Data 6. For the double mutant *PIK3CA* E545K and *ERBB2* L755S assay, a primer/probe master mix was prepared, mixing stock primers and probes at 100 μM each to a concentration of 18 μM in 5 μL per sample. 5 μL of this mix was combined with 45 μL 1:2 diluted isolated cfDNA and 50 μL ddPCR Supermix for Probes (No dUTP) (Biorad). This solution was distributed into cartridges and formed into droplets using the QX200 droplet generator (Biorad). PCR was performed according to the manufacturer’s protocol, and results were read using the QX200 droplet reader (Biorad).

### Statistics and reproducibility

GraphPad Prism 9.5.0 (La Jolla, CA) was used to generate all graphs with statistics indicated. All experiments were performed at least twice independently and performed in at least three biological replicates, as indicated in their respective figure legends. Data shown in bar graph form are means ± SEM aside from Supplementary Figs. 2 and 3, which show medians and box and whisker plots respectively. All comparisons of two groups were performed through student’s *t* test, while comparisons of three or more were analyzed through ANOVA. For ANOVA, Dunnett’s tests were used to compare the control group to all other groups or Sidak’s test to compare specific groups. All tests were parametric aside from in Supplementary Fig. S2, which leveraged non-parametric analyses due to imbalance in group size. Correlation analyses were performed in R (Posit) using the Pearson correlation. Outliers were removed from correlation analyses using the ROUT method at the most stringent *Q* value of 0.1%. A *P* value of less than 0.05 was considered significant for all studies and is included in each graph where statistics were performed. Specific statistical analysis for each experiment is described in its figure legend.

### Reporting summary

Further information on research design is available in the Nature Portfolio Reporting Summary linked to this article.

### Supplementary information

Supplemental Figures

Description of Additional Supplementary Files

Supplementary Data 1-6

Supplementary Data 7

Reporting Summary

## Data Availability

Next-generation sequencing data generated for our CRISPR screen has been deposited at Dryad, and will be publicly available as of the date of publication (10.5061/dryad.k0p2ngfd2), also listed in Supplementary Data 6. Source data for bar graphs can be found in Supplementary Data 7. Original western blot images for all figures are included as Supplementary Figs. S12-S18. This paper does not report the original code. All original CRISPR cell lines generated for this paper can be requested from the corresponding author. All other lines can be found through ATCC. Any additional information required to reanalyze the data reported in this work paper is available from the lead contact, Ben Ho Park (ben.h.park@vumc.org).
