# Supplementary material for: An in vitro CRISPR screen of cell-free DNA identifies apoptosis as the primary mediator of cell-free DNA release
Source: Commun Biol. 2024 Apr 10;7:441. doi: 10.1038/s42003-024-06129-1 (PMC11006667; doi:10.1038/s42003-024-06129-1)
Supplement: Supplementary file 1 — Supplemental Figures [file 42003_2024_6129_MOESM1_ESM.pdf]

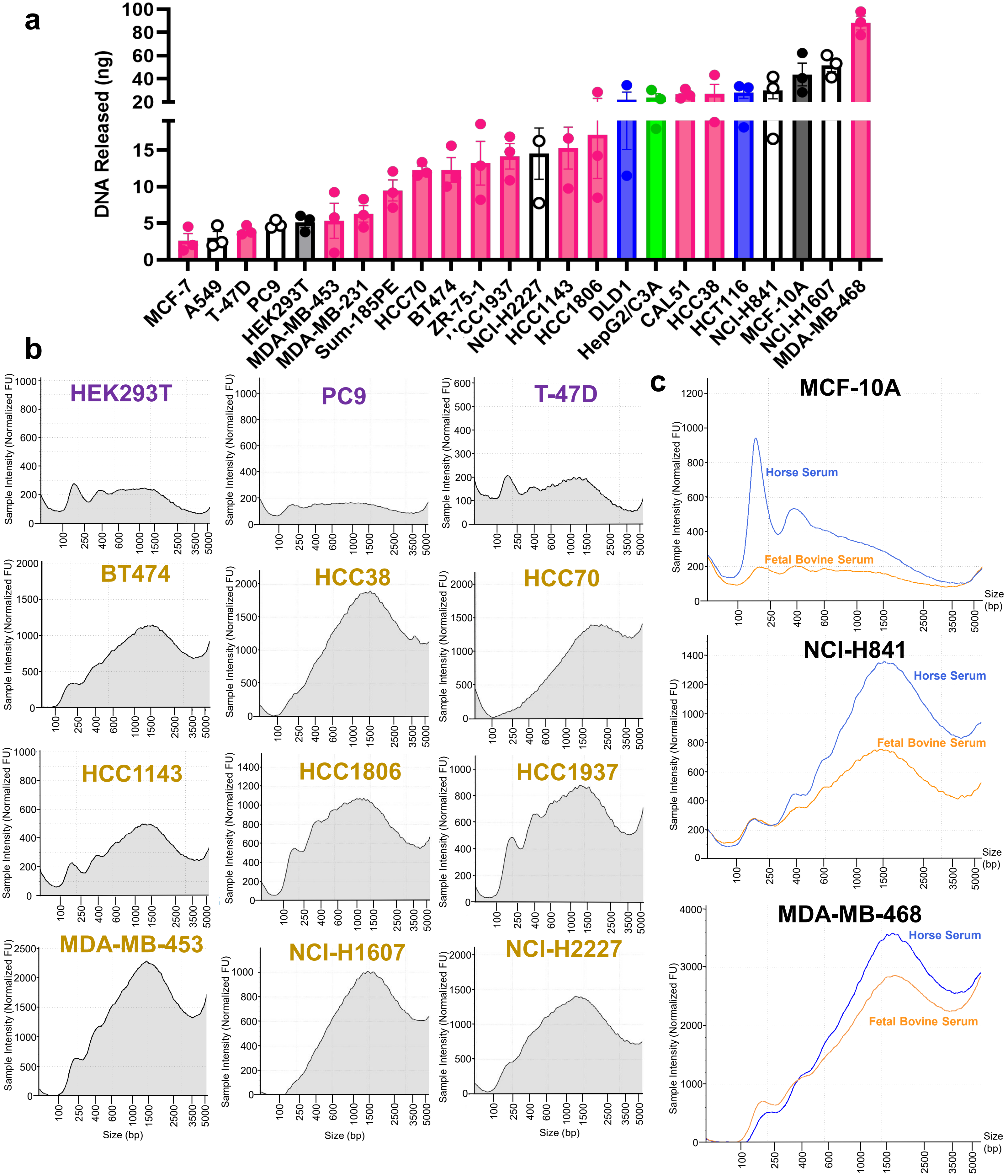

Supplementary Figure S1. Additional information from cell-free DNA cell line panel. a Quantification of raw values of cfDNA release from cell lines in culture. Data represent mean fold change ± SEM in absolute DNA release, n=3 biologically independent samples for each cell line. **b** Fragmentation patterns of additional cell lines from the cfDNA panel. Cell lines HEK293T, PC9, and T-47D (purple text) are representative of cell lines with a left skew, all other depicted cell lines (yellow text) are representative of cell lines with a right skew. Representative traces shown, of traces individually run at least n=3 times . **c** Fragmentation patterns of cell lines treated with different serum conditions. Blue traces represent horse serum treatment, whereas orange represent fetal bovine serum. Electropherograms were individually run at least n=3 times and representative traces were selected.

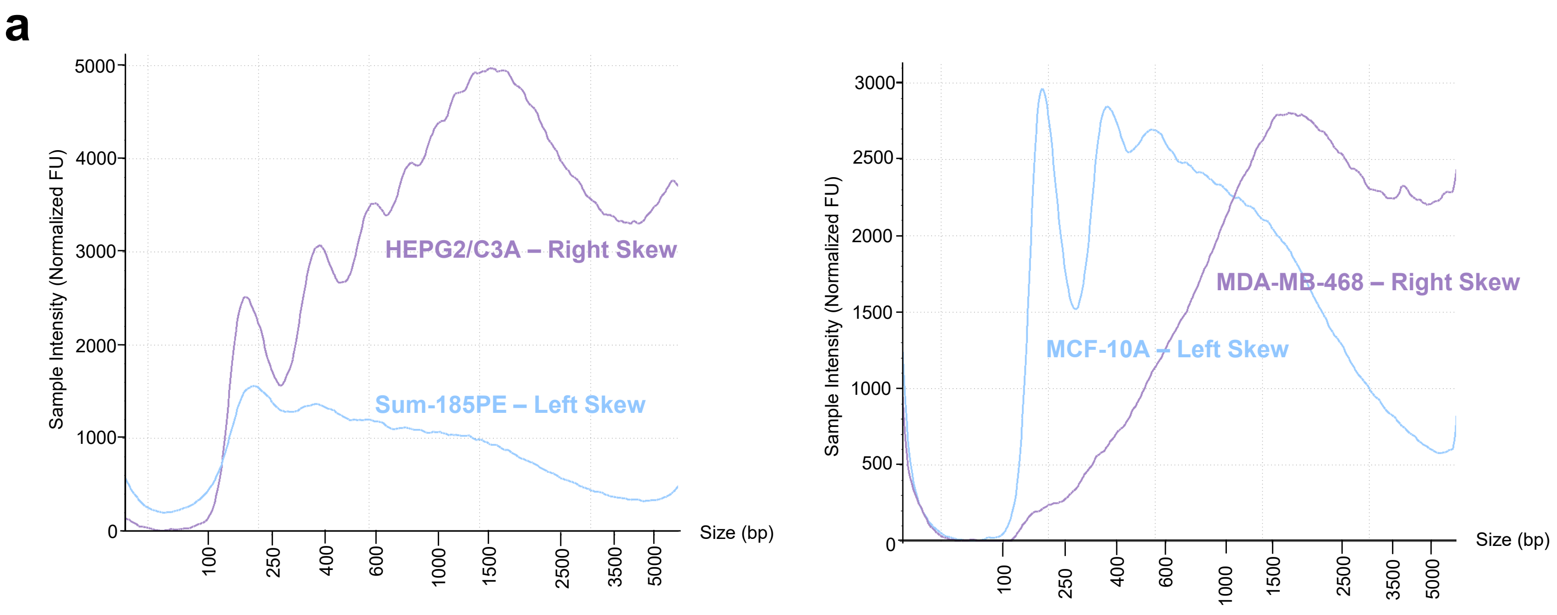

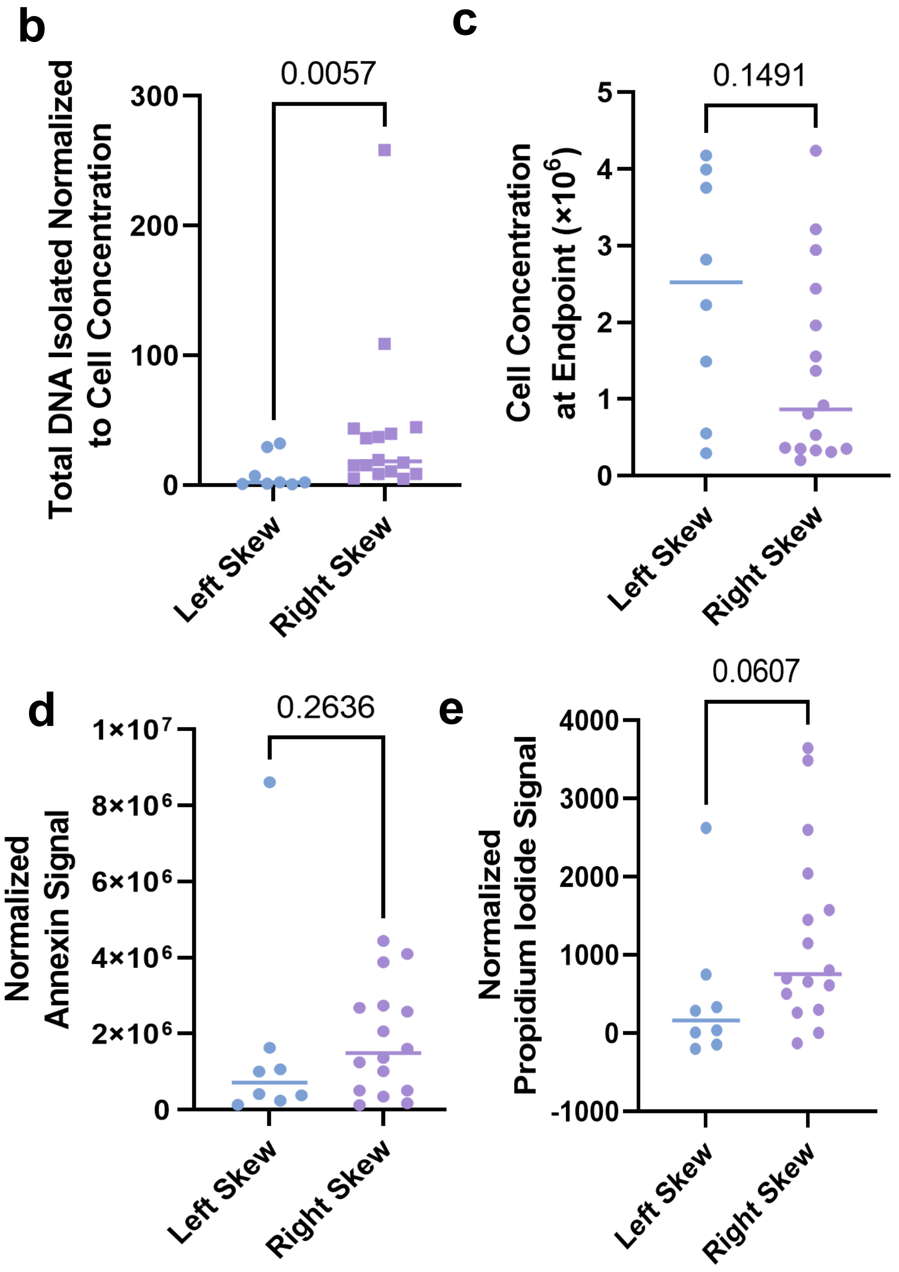

Supplementary Figure S2. Comparison of cell lines with left and right skewing of cell-free DNA.
a Fragmentation patterns of example left-skewing and right-skewing cell lines overlapped to indicate their different fragmentation pattern. In the left panel, left-skewed Sum-185PE and right-skewed HEPG2/C3A are reproduced as an overlay from Figure 1F. In the right panel, example fragmentation patterns from left-skewed MCF-10A is overlapped with right-skewed MDA-MB-468. **b** Quantification of total DNA release from left-skewed and right-skewed cell lines. Data represent mean DNA release normalized to cell concentration from Figure 1E. Bar line is median, n=8 for left-skew and 16 for right-skew. **c** Quantification of proliferation from left-skewed and right-skewed cell lines. Data represent mean cell concentration after seeding at equivalent starting concentrations used to normalized cfDNA release in Figure 1E. Bar line is median, n=8 for left-skew and 16 for right-skew. **d** Quantification of Annexin V signal from left-skewed and right-skewed cell lines. Data represent mean Annexin V normalized to cell concentration from Figure 5A. Bar line is median, n=8 for left-skew and 16 for right skew. **e** Quantification of Propidium Iodide signal from left-skewed and right-skewed cell lines. Data represent mean Propidium Iodide signal normalized to cell concentration from Figure 5B. Bar line is median, n=8 for left-skew and 16 for right skew. Mann-Whitney U test was run due the large discrepancy in total number of left-skewed vs. right-skewed cell lines (8 vs. 16) and skewing.

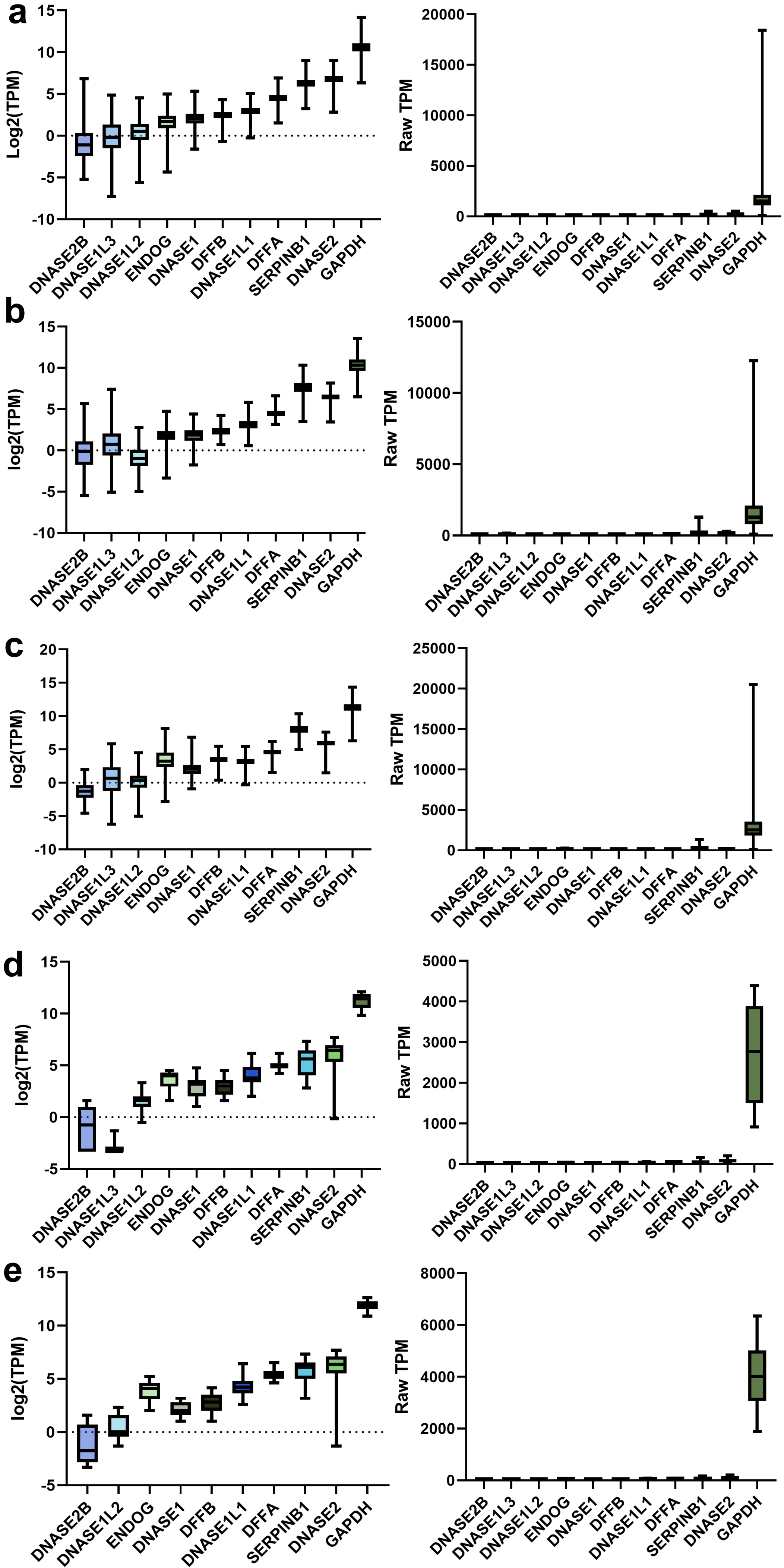

Supplementary Figure S3. Expression of DNAses by TCGA and cell line cohorts. a Raw TPM and Log2 transformed TPMs of the expression of DNAses in the TCGA breast cancer cohort (n=1230). **b** Raw TPM and Log2 transformed TPMs of the expression of DNAses in the TCGA lung adenocarcinoma cohort (n=600) **c** Raw TPM and Log2 transformed TPMs of the expression of DNAses in the TCGA colorectal cancer cohort (n=522). **d** Raw TPM and Log2 transformed TPMs of the expression of DNAses in the Cancer Cell Line atlas including all available cell lines used in this study (n=6 for *DNASE1L3*, n=7 for *DNASE2B*, and n=17 for all others). **e** Raw TPM and Log2 transformed TPMs of the expression of DNAses in the Genetech RNA-seq database including all available cell lines used in this study (n=1 for *DNAse1L3*, 5 for *DNASE2B*, and 13 for all others). For all box and whisker plots, center line, median; box limits, upper and lower quartiles; whiskers, 1.5x interquartile range.

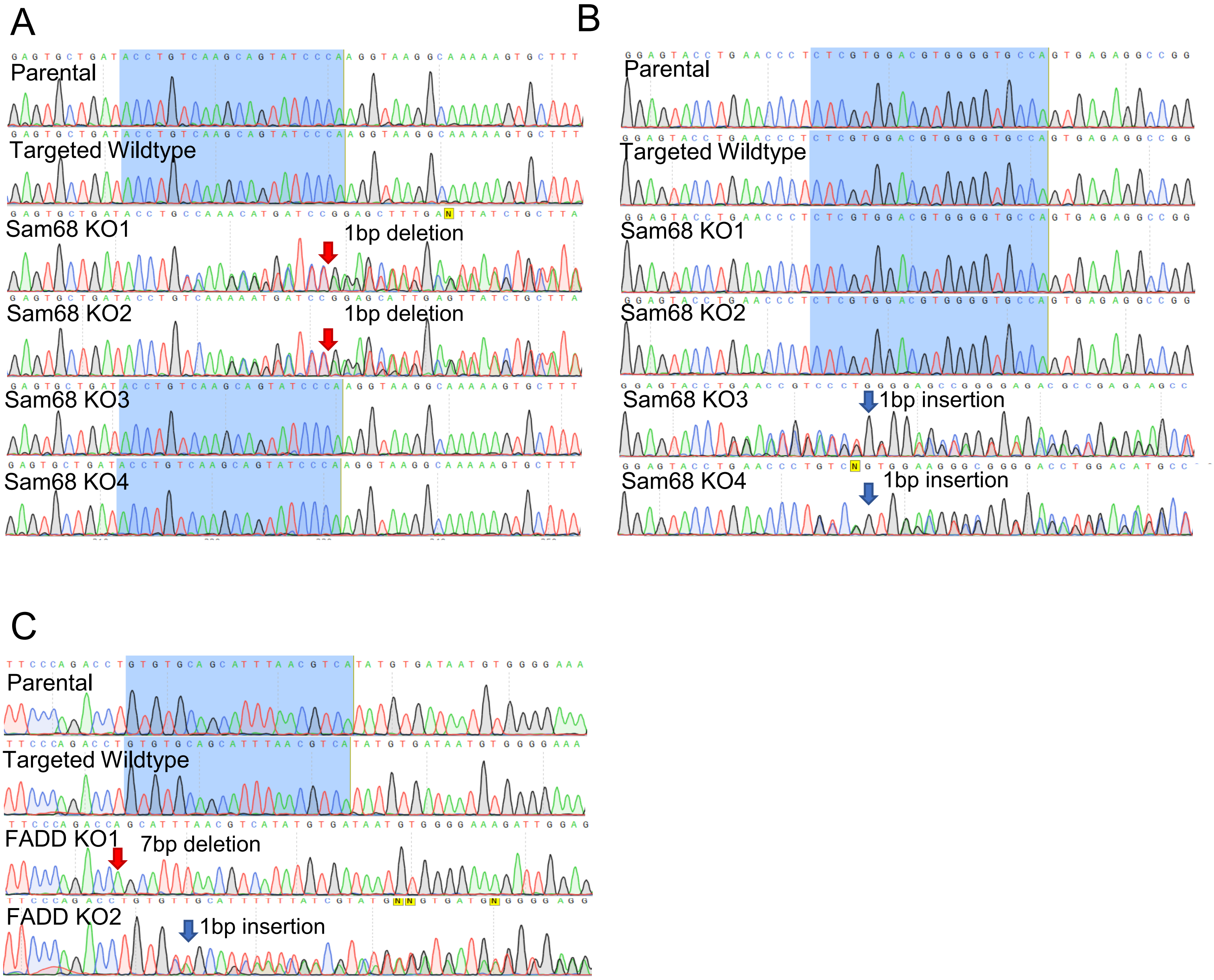

Supplementary Figure S4. Gene sequence of Sam68 and FADD knockout cell lines in MCF-10A background. a Sequence of Sam68 clones at the cut-site of Sam68_sgRNA_1, with sgRNA sequence highlighted in blue. **b** Sequence of Sam68 clones at the cut-site of Sam68_sgRNA_2, with sgRNA sequence highlighted in blue. **c** Sequence of FADD clones at the cut-site of FADD_sgRNA_1, with sgRNA sequence highlighted in blue.

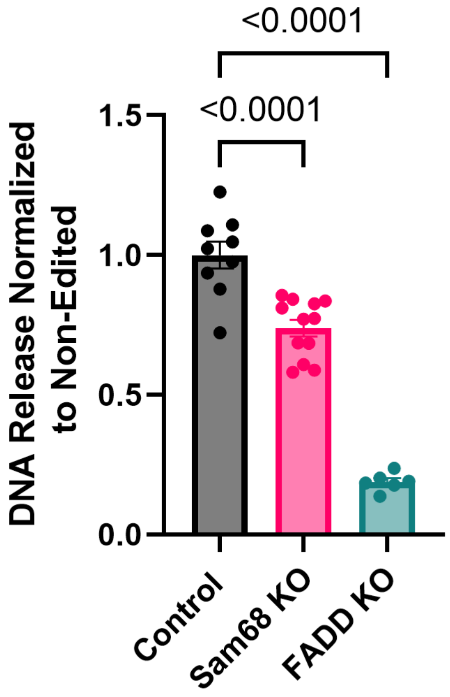

Supplementary Figure S5. Collapsed quantification of DNA release from MCF-10A KO cell lines in culture. Parental cells and TWT cells were averaged and labeled control. All four Sam68 KO and both FADD KO cell lines are respectively grouped. Data represent mean fold change ± SEM internally normalized to cell concentration for each cell line and then normalized to control; n=3 independent biological samples for each cell line before combining. Statistics from ANOVA with Dunnett’s post-hoc test.

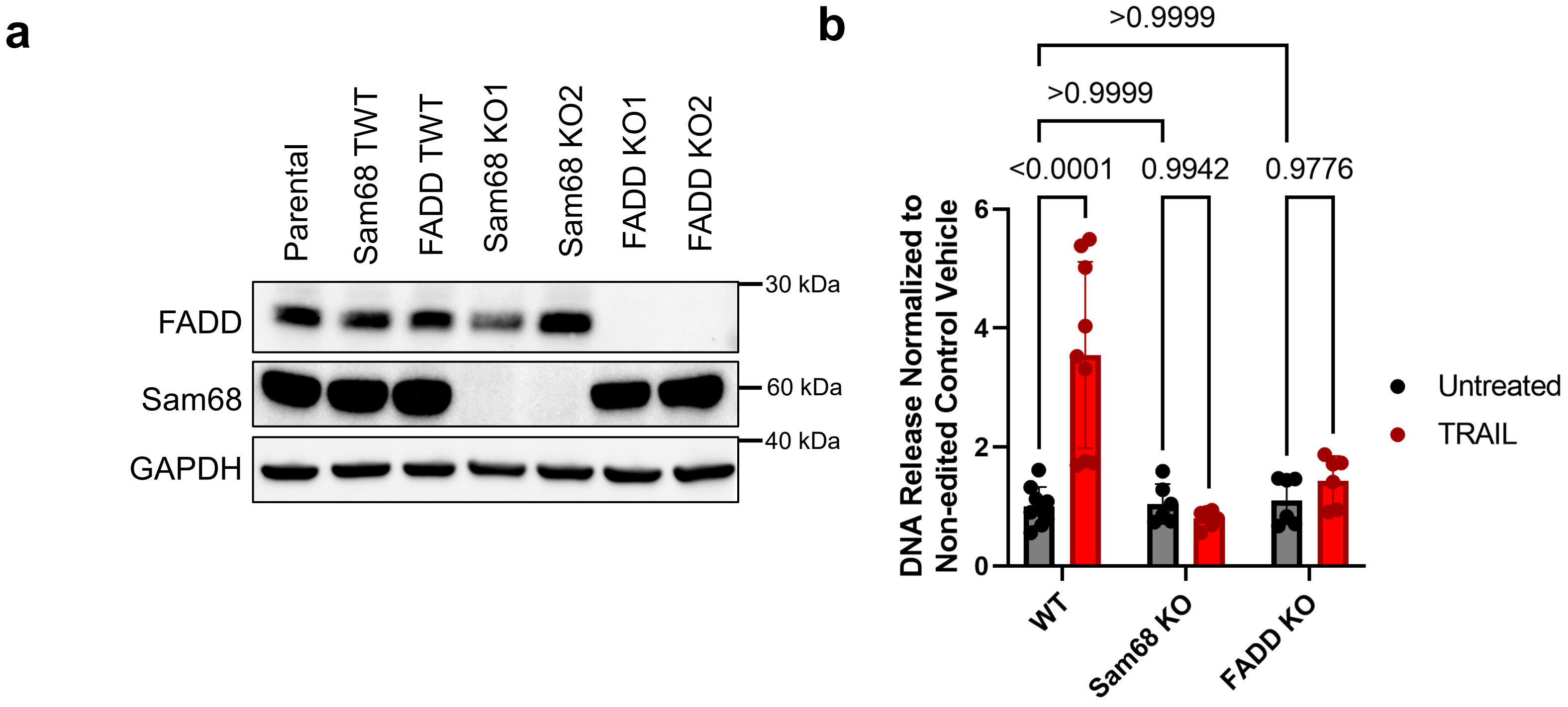

Supplementary Figure S6. Manipulation of MCF-10A screen hits in non-MCF-10A backgrounds. **a** Immunoblot analysis of Sam68 and FADD after CRISPR-mediated double knockout in the MDA-MB-468 background. **b** Quantification of DNA release from MDA-MB-468 KO cell lines in culture. Parental cells and TWT cells were averaged and labeled control. Both Sam68 KO and FADD KO knockout lines are respectively grouped. Data represent mean fold change ± SEM internally normalized to cell concentration for each cell line and then normalized to parental MDA-MB-468; n=3 independent biological samples for each cell line before combining.

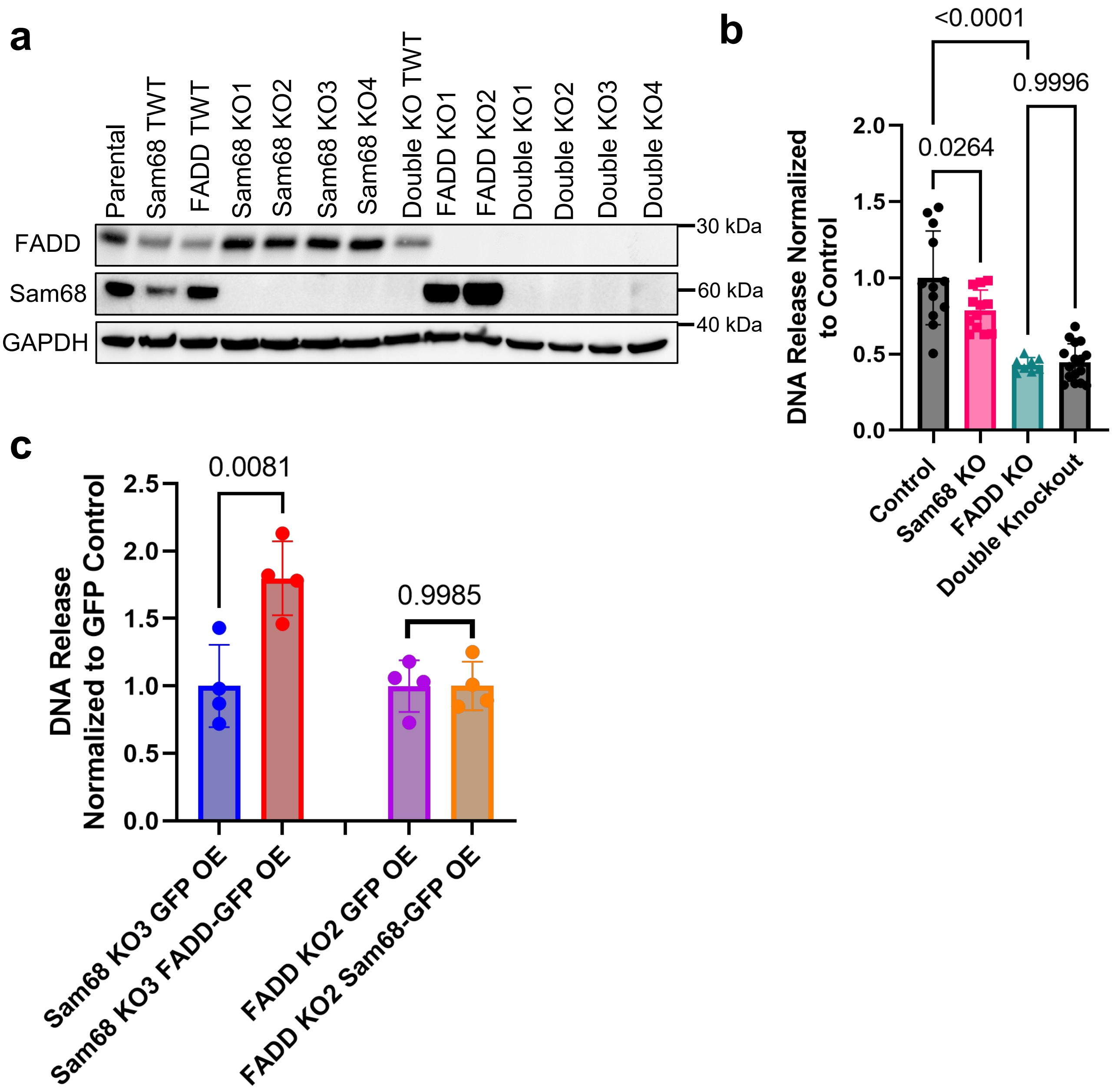

Supplementary Figure S7. Manipulation of Sam68 and FADD in MCF-10A background indicates shared pathway. **a** Immunoblot analysis of Sam68 and FADD after CRISPR-mediated double knockout in the MCF-10A background. **b** Quantification of DNA release from MCF-10A KO and double KO cell lines in culture. Parental cells and TWT cells were averaged and labeled control. All four Sam68 KO, both FADD KO, and all four double knockout lines initially derived from Sam68 KO3 and Sam68 KO4 are respectively grouped. Data represent mean fold change ± SEM internally normalized to cell concentration for each cell line and then normalized to parental MCF-10A; n=4 biologically independent samples for each cell line before combining. Compared statistically by ANOVA with Dunnett's post-hoc test. **c** Quantification of DNA release from MCF-10A KO and over-expression lines. One Sam68 and one FADD KO line were infected with either GFP-only lentivirus, or a GFP-tagged version of the opposing protein. Data represent mean fold change ± SEM internally normalized to cell concentration for each cell line and then normalized to GFP-expressing control; n=4 biologically independent samples for each cell line before combining. Compared statistically by student's t-test.

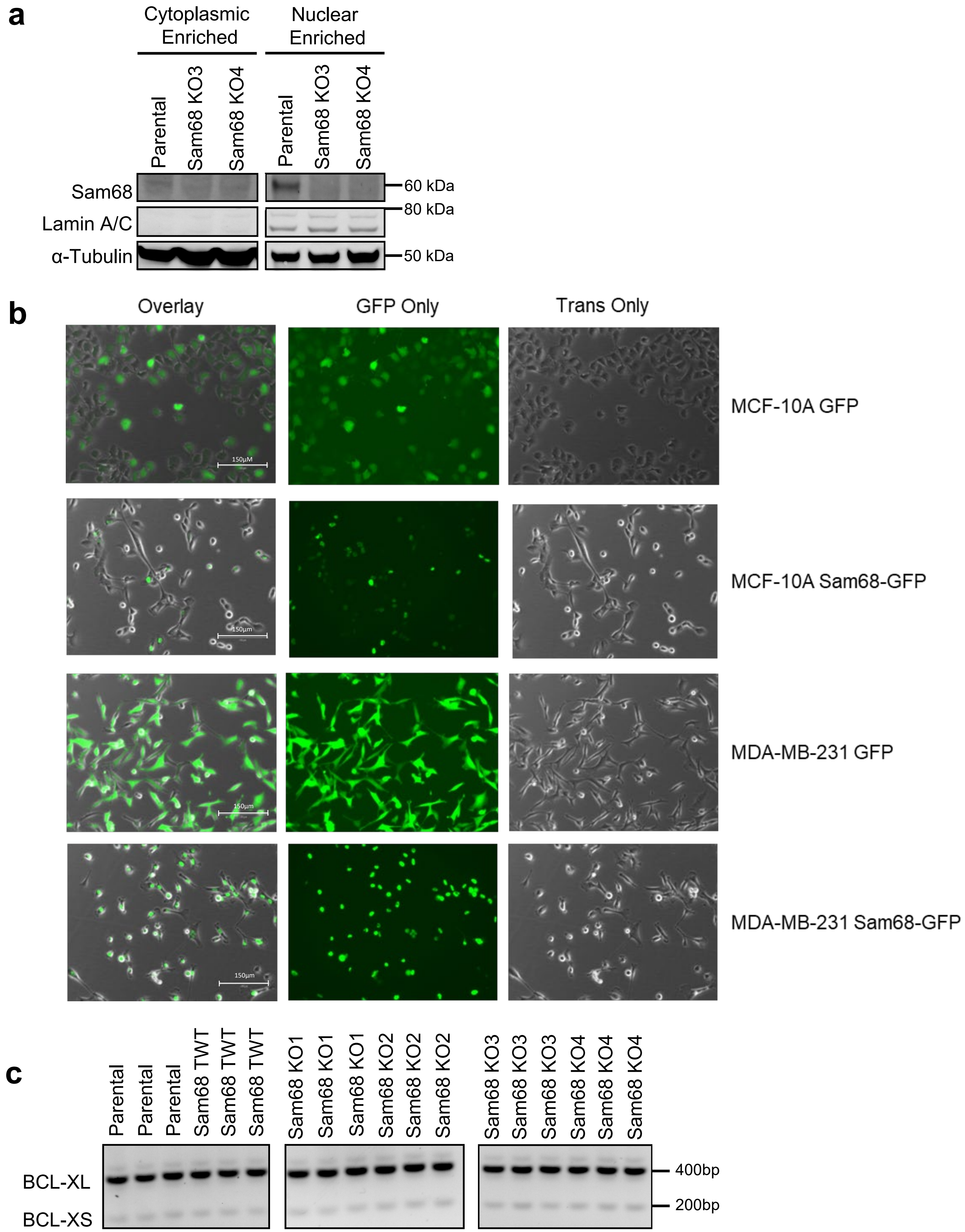

Supplementary Figure S8. Sam68 modulates cell-free DNA release likely through roles within the nucleus. a Immunoblot analysis of Sam68 in nuclear and cytoplasmic enriched fractions of MCF-10A Sam68 KO cells. **b** Localization of GFP or GFP-tagged Sam68 in MCF10A and MDA-MB-231 cells. Scale bar is 150µm. **c** Splicing of BCL-X into BCL-XL and BCL-XS in MCF-10A Sam68 KO cell lines.

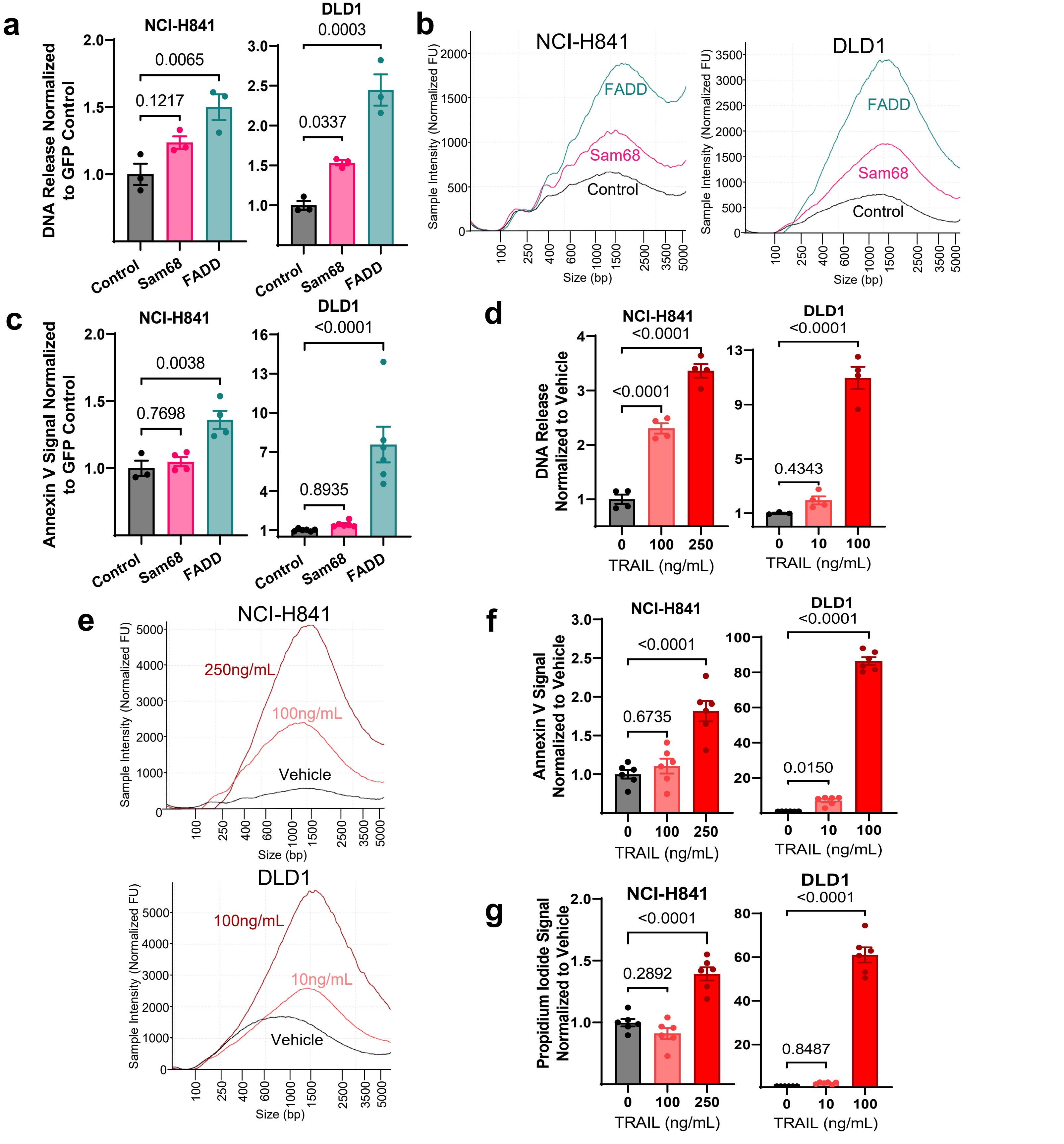

Supplementary Figure S9. Additional validation of Sam68, FADD, and TRAIL as regulators of cfDNA release in cancer cell lines. **a** Quantification of DNA release from NCI-H841 and DLD1 cancer cell lines with overexpression of GFP-tagged Sam68 or FADD. Data represent mean fold change ± SEM in DNA release, normalized to cell concentration for each cell line then overall to GFP control; n=3 biologically independent samples. **b** Fragmentation pattern of NCI-H841 and DLD1 cancer cell lines with overexpression of GFP-tagged Sam68 or FADD. Electropherograms were individually run at least n=3 times and representative traces were selected. **c** Quantification of Annexin V signal from NCI-H841 and DLD1 cancer cell lines with overexpression of GFP-tagged Sam68 or FADD. Data represent mean fold change ± SEM RLU signal normalized to cell concentration for each cell line, then overall to GFP control; n=3 biologically independent replicates for NCI-H841 control and 4 for KOs, with n=6 for all DLD1 conditions. **d** Quantification of DNA release from NCI-H841 and DLD1 cancer cell lines treated with TRAIL ligand. Data represent mean fold change ± SEM in DNA release normalized to cell concentration at collection for each cell line then overall to GFP control; n=3 biologically independent samples for DLD1 control, n=4 for all other conditions. **e** Fragmentation pattern of NCI-H841 and DLD1 cancer cell lines treated with TRAIL ligand. Electropherograms were individually run at least n=3 times and representative traces were selected. **f** Quantification of Annexin V signal from NCI-H841 and DLD1 cancer cell lines treated with TRAIL Ligand. Data represent mean fold change ± SEM in signal (RLU) normalized to cell concentration at collection for each cell line, then overall to vehicle control; n=6 biologically independent samples. **g** Quantification of Propidium Iodide signal from NCI-H841 and DLD1 cancer cell lines treated with TRAIL Ligand. Data represent mean fold change ± SEM in signal (RFU) normalized to cell concentration at collection for each cell line, then overall to vehicle control; n=6 biologically independent samples. All statistics were ANOVA with Dunnett's multiple comparison test at endpoint.

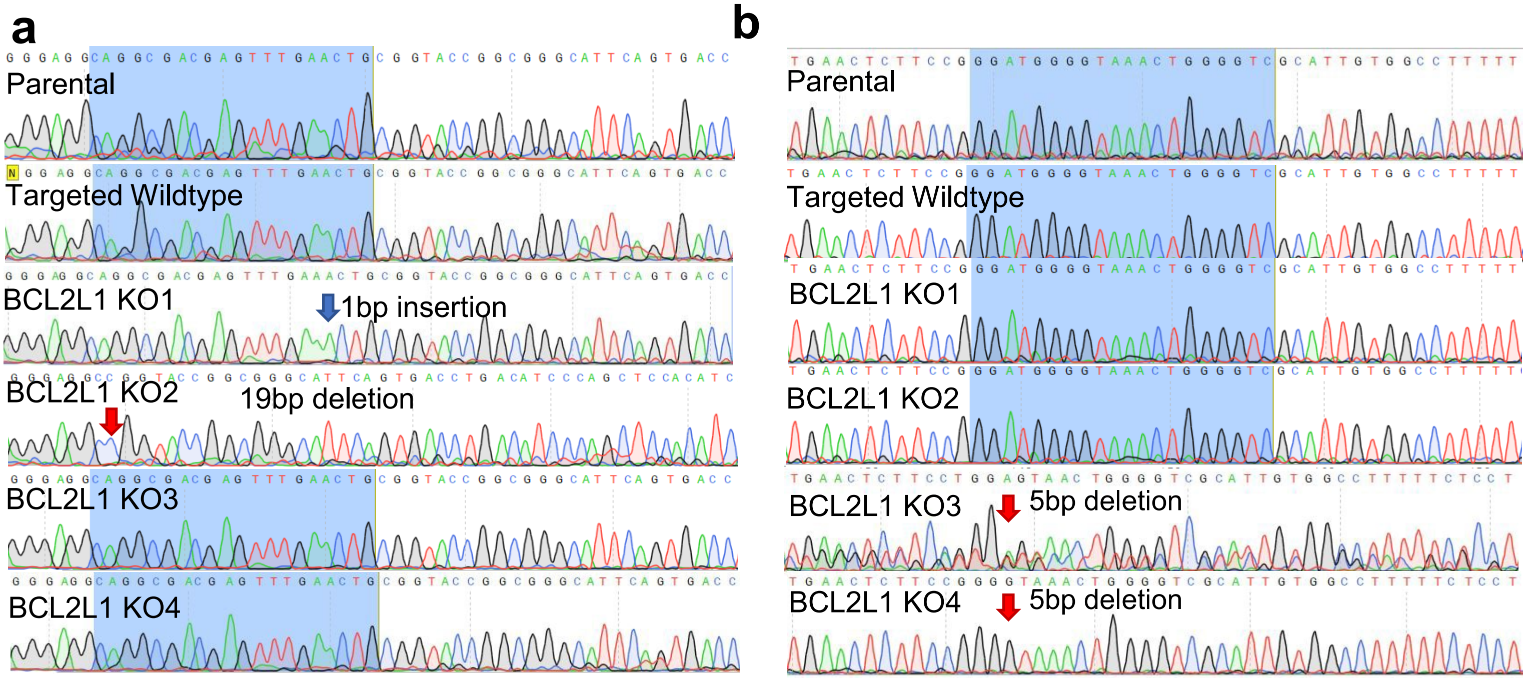

Supplementary Figure S10. Gene sequence of *BCL2L1* knockout cell lines. a Sequence of A549 *BCL2L1* knockout clones at the cut-site of BCL2L1_sgRNA_1, with sgRNA sequence highlighted in blue. **b** Sequence of A549 *BCL2L1* knockout clones at the cut-site of BCL2L1_sgRNA_2, with sgRNA sequence highlighted in blue.

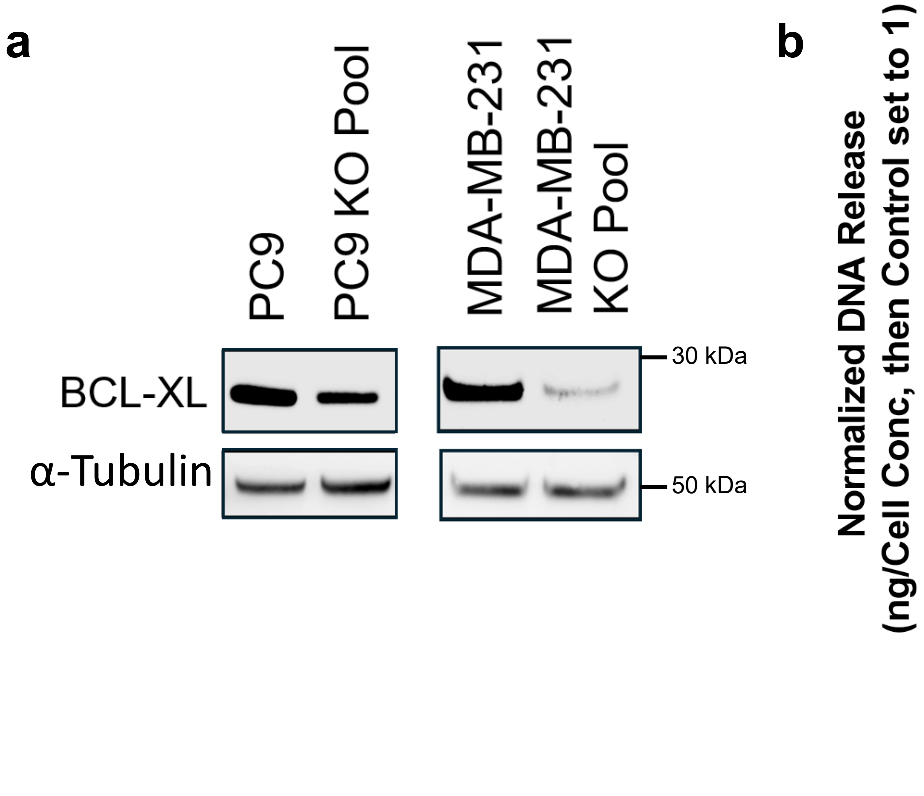

Supplementary Figure S11. Manipulation of BCL2L1 in additional cell lines. **a** Immunoblot analysis of BCL-XL in CRISPR knockout pooled cell lines in the PC9 and MDA-MB-231 cell lines. **b** Quantification of DNA release from pooled KO cell lines in culture. Control cells are parental cells treated only with Cas9. Data represent mean fold change ± SEM internally normalized to cell concentration for each cell line and then normalized to control for each line; n=3 biologically independent samples for each cell line or cell pool.

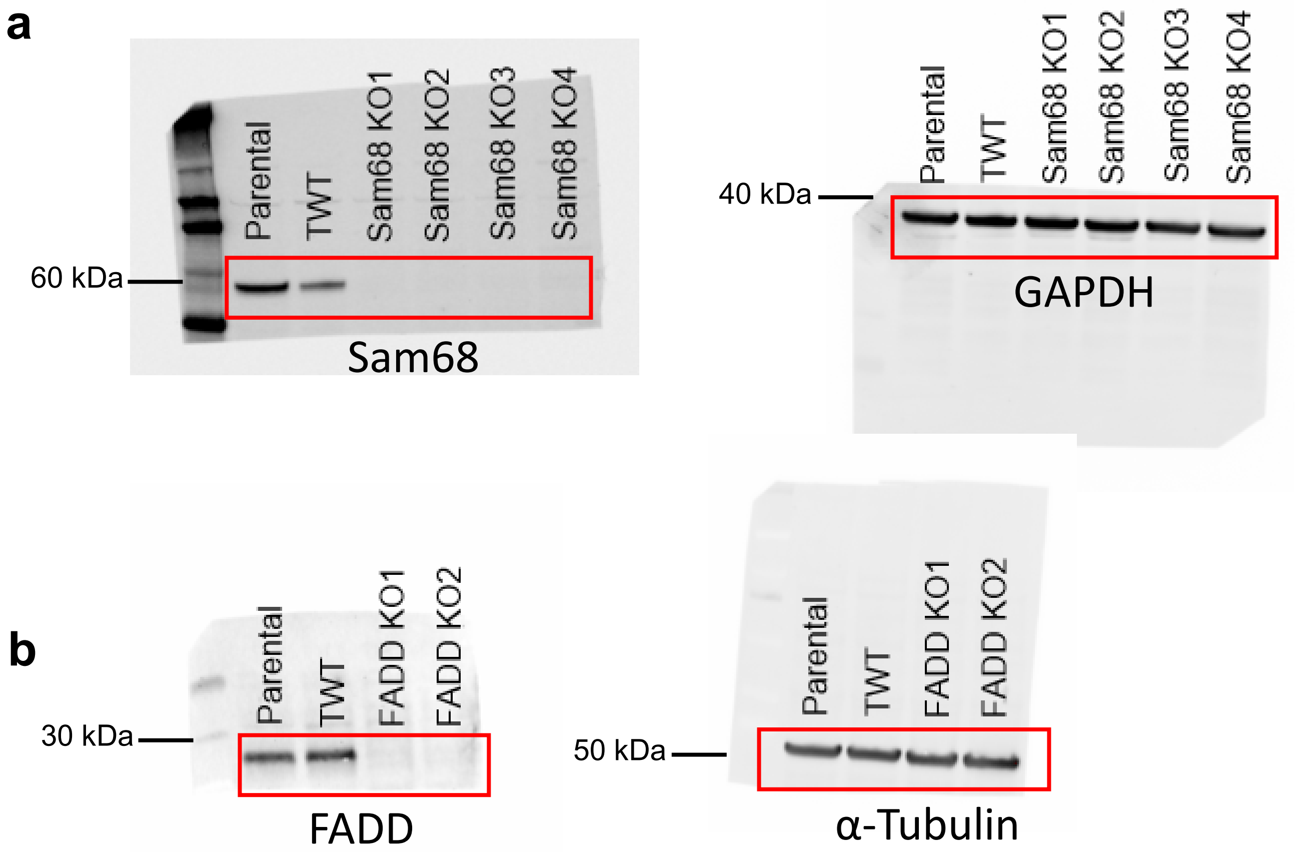

Supplementary Figure S12. Uncropped and unedited blots for Figure 3a. **a** Blot for Sam68 using Polyclonal Mouse Sam68 Antibody (7-1, Santa Cruz sc-1238) with Digital anti-mouse-HRP secondary (KwikQuant R1005). Blot for GAPDH using Monoclonal Rabbit GAPDH Antibody (D16H11, Cell Signaling Technology 5174) with Goat anti-Rabbit IgG (H+L) Alexa Fluor Plus 647 (ThermoFisher A32733). **b** Blot for FADD using Monoclonal Rabbit FADD (EPR4415, Abcam ab108601) and blot for α-Tubulin using Polyclonal Rabbit α-Tubulin (Abcam ab4074) both with anti-Rabbit IgG (H+L) Alexa Fluor Plus 647 (ThermoFisher A32733).

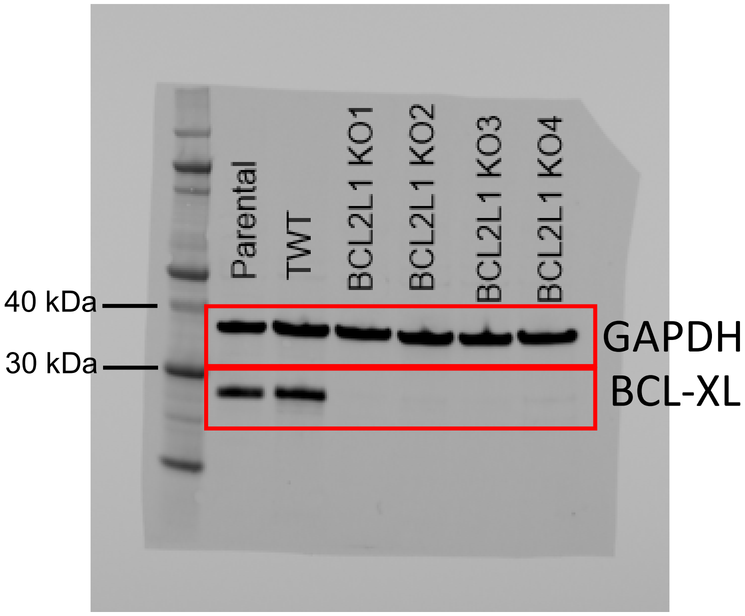

Supplementary Figure S13. Uncropped and unedited blots for Figure 5g. Blot for Gapdh using Monoclonal Rabbit GAPDH Antibody (D16H11, Cell Signaling Technology 5174) and blot for Monoclonal Rabbit BCL-XL (54H6, Cell Signaling Technology 2764) with Goat anti-Rabbit IgG (H+L) Alexa Fluor Plus 647 secondary (ThermoFisher A32733) run simultaneously.

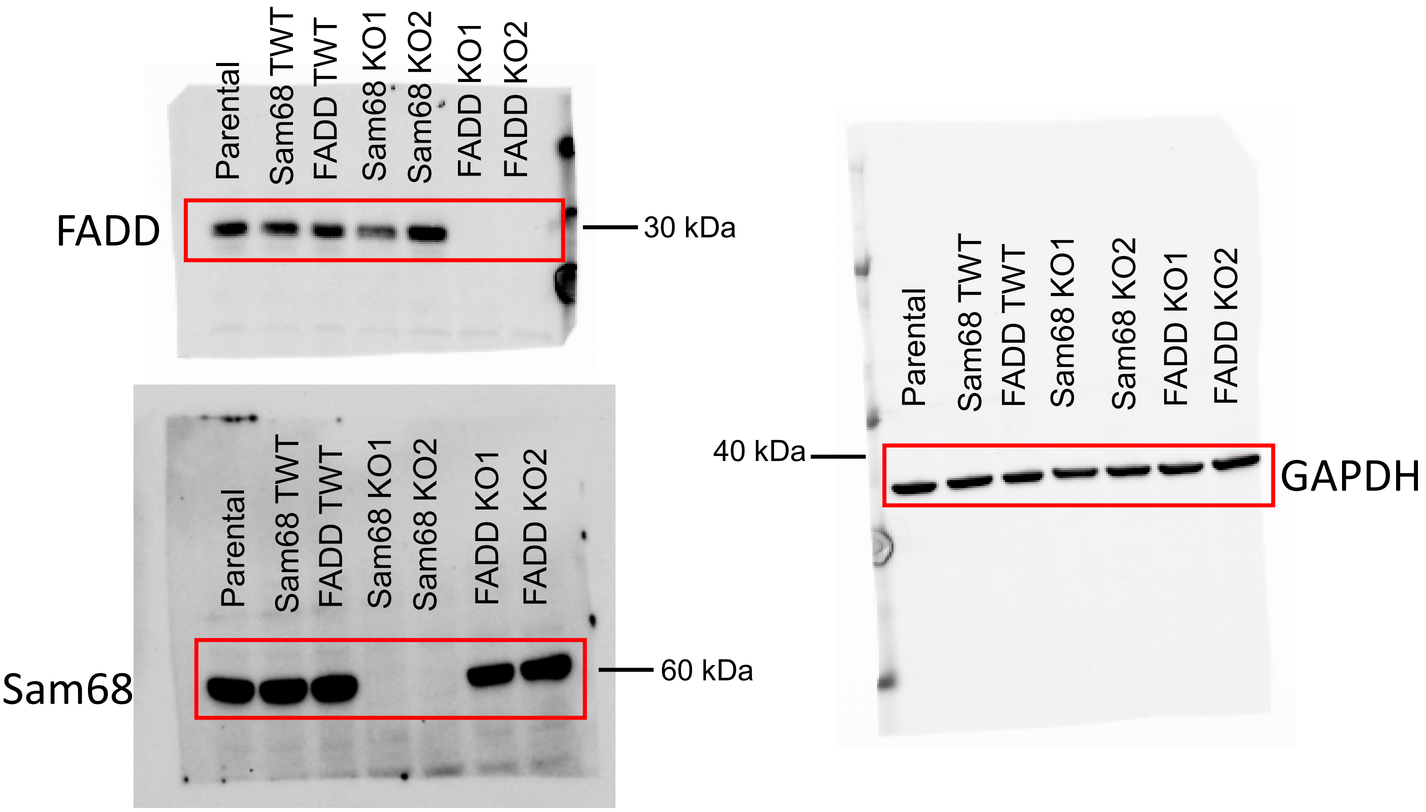

Supplementary Figure S14. Uncropped and unedited blots for Supplementary Figure S6a. Blot for blot for FADD using Monoclonal Rabbit FADD (EPR4415, Abcam ab108601) and GAPDH using Monoclonal Rabbit GAPDH Antibody (D16H11, Cell Signaling Technology 5174) with Goat anti-Rabbit IgG (H+L) Alexa Fluor Plus 647 secondary (ThermoFisher A32733). Blot for Sam68 using Polyclonal Mouse Sam68 Antibody (7-1, Santa Cruz sc-1238) with Digital anti-mouse-HRP secondary (KwikQuant R1005). GAPDH run on separate blot using same lysates at same volumes due to size similarity with FADD.

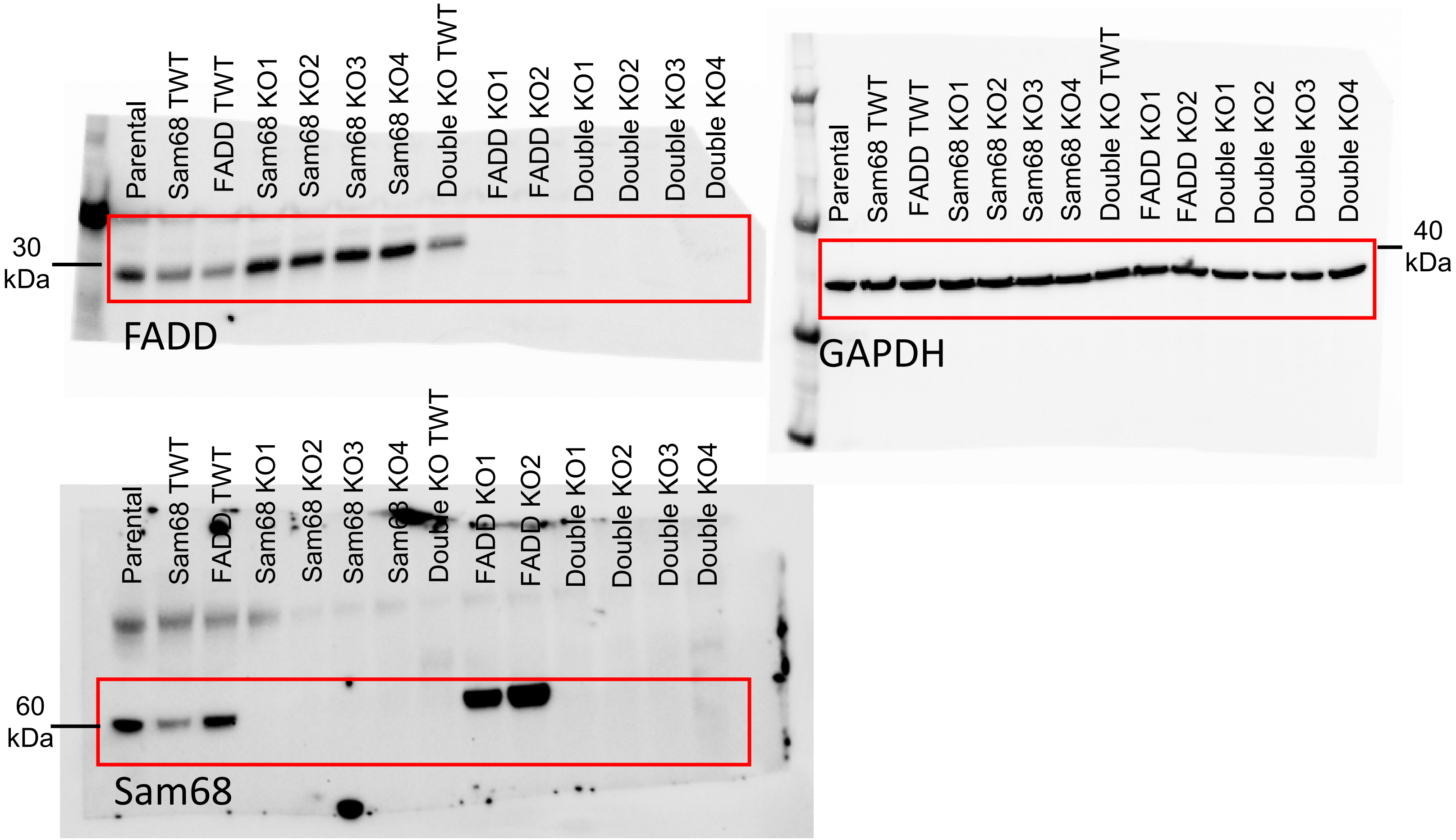

Supplementary Figure S15. Uncropped and unedited blots for Supplementary Figure S7a. Blot for FADD using Monoclonal Rabbit FADD (EPR4415, Abcam ab108601) and blot for Gapdh using Monoclonal Rabbit GAPDH Antibody (D16H11, Cell Signaling Technology 5174) with Goat anti-Rabbit IgG (H+L) Alexa Fluor Plus 647 secondary (ThermoFisher A32733). Blot for Sam68 using Polyclonal Mouse Sam68 Antibody (7-1, Santa Cruz sc-1238) with Digital anti-mouse-HRP secondary (KwikQuant R1005). GAPDH run on separate blot using same lysates at same volumes due to size similarity with FADD.

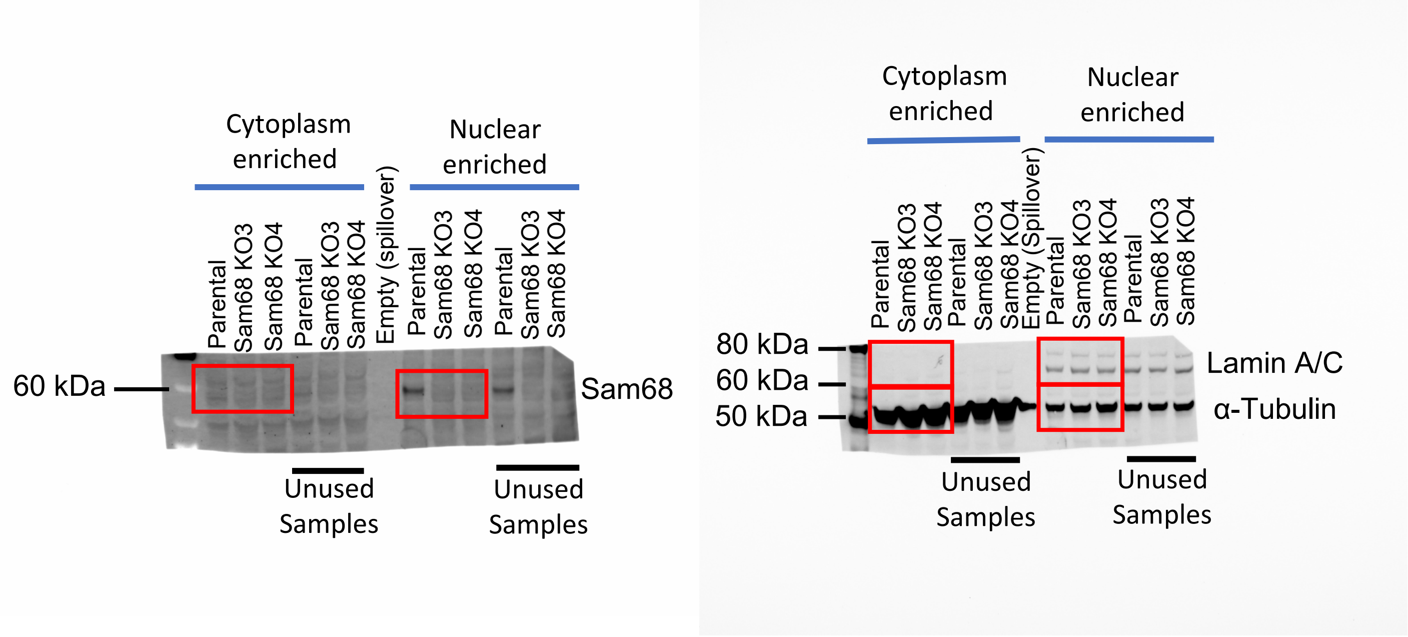

Supplementary Figure S16. Uncropped and unedited blots for Supplementary Figure S8a. Blot for a-tubulin using Polyclonal Rabbit a-Tubulin (Abcam ab4074) and Polyclonal Rabbit Lamin A/C (Cell Signaling Technology 2032) simultaneously with Goat anti-Rabbit IgG (H+L) Alexa Fluor Plus 647 (ThermoFisher A32733) secondary. Sam68 detected on same blot using Polyclonal Mouse Sam68 Antibody (7-1) Santa Cruz sc-1238 with Goat anti-Mouse IgG (H+L) Alexa Fluor 488 (ThermoFisher A11029).

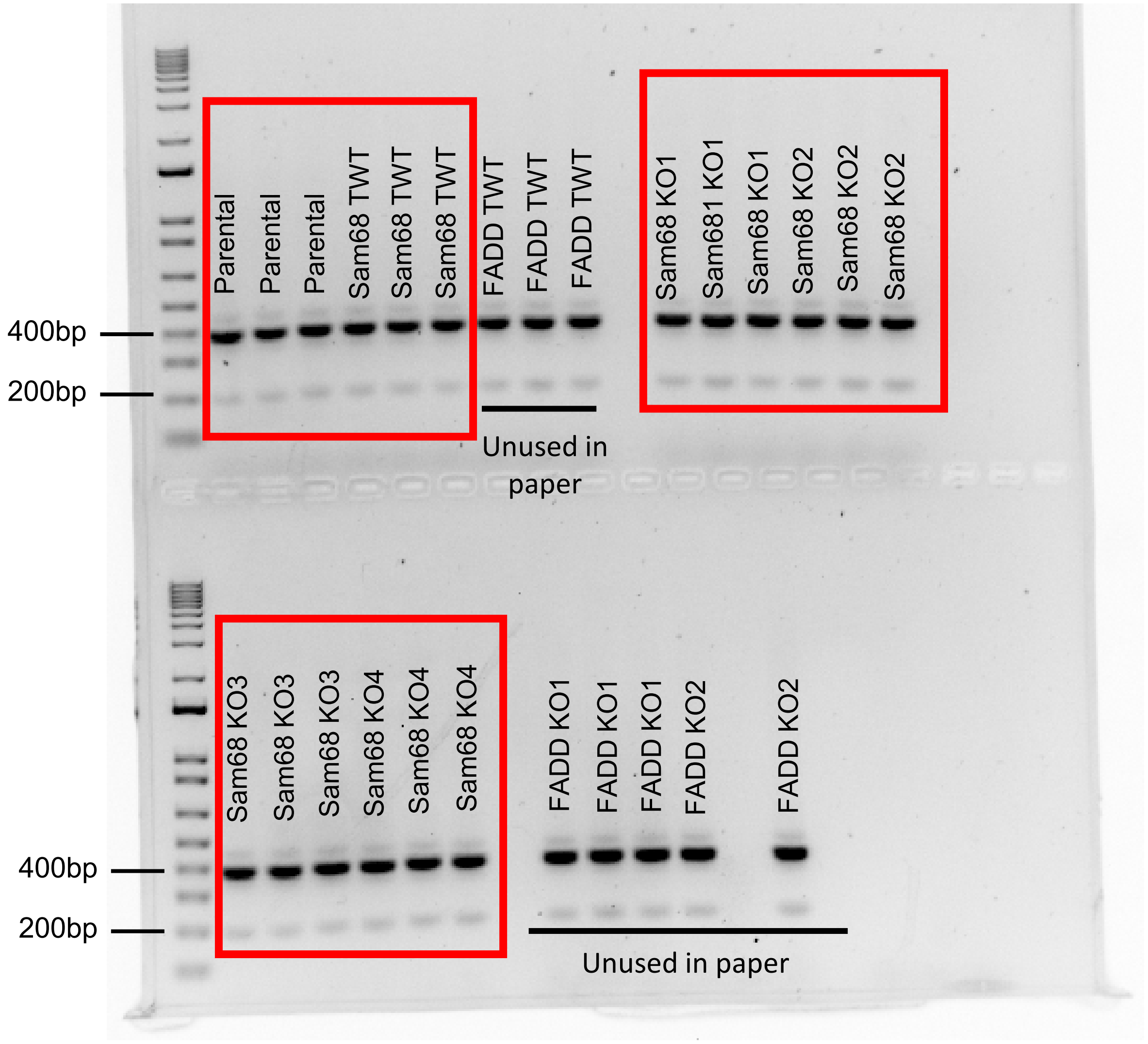

Supplementary Figure S17. Uncropped and unedited gel for Supplementary Figure S8c. RT-PCR of MCF-10A Sam68 knockout *BCL2L1* transcripts.

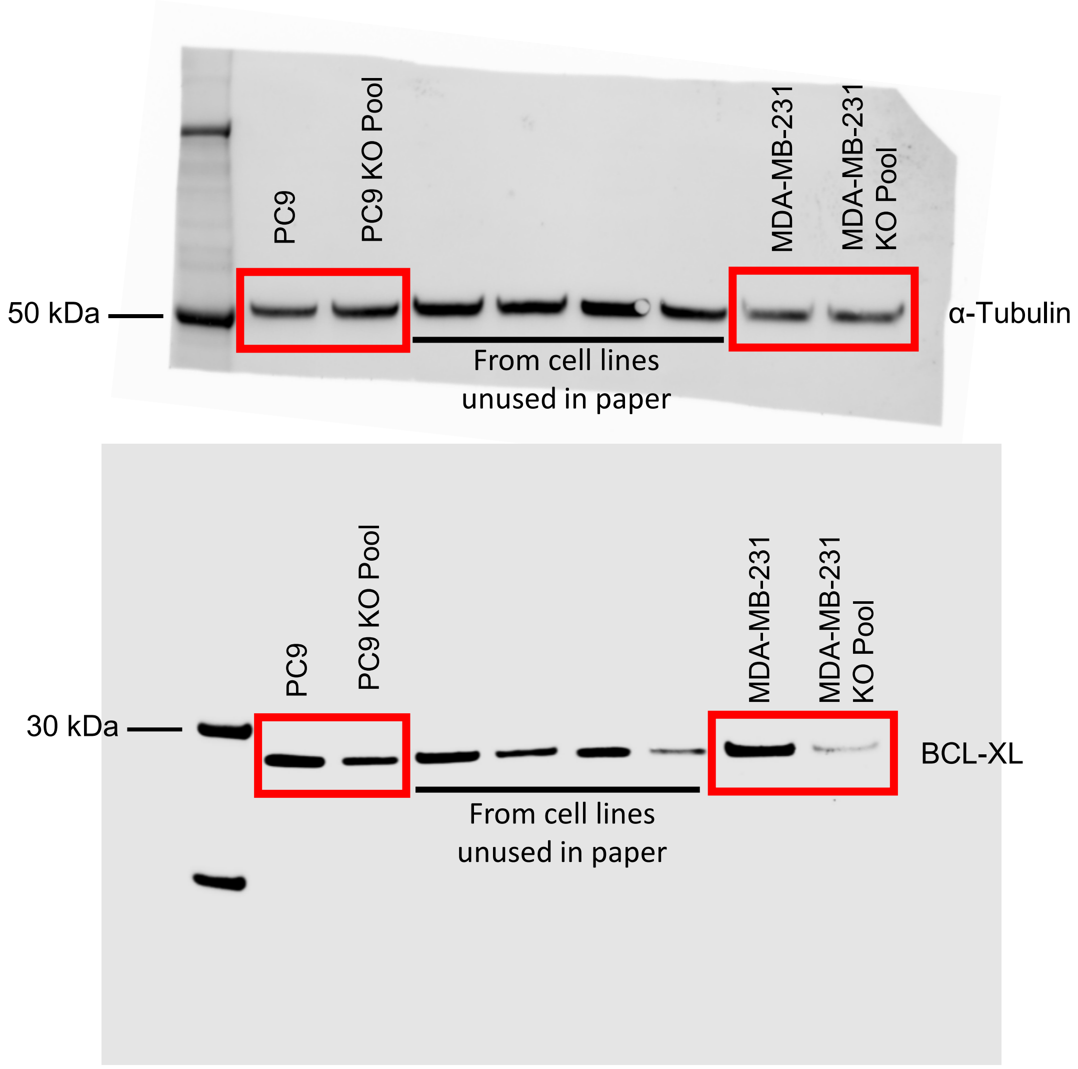

Supplementary Figure S18. Uncropped and unedited blots for Figure S11a. Blot for α-Tubulin using Polyclonal Rabbit a-Tubulin Abcam ab4074 and blot for Monoclonal Rabbit BCL-XL (54H6, Cell Signaling Technology 2764) with Goat anti-Rabbit IgG (H+L) Alexa Fluor Plus 647 secondary (ThermoFisher A32733).
