# Supplementary material for: An in vitro CRISPR screen of cell-free DNA identifies apoptosis as the primary mediator of cell-free DNA release
Source: Commun Biol. 2024 Apr 10;7:441. doi: 10.1038/s42003-024-06129-1 (PMC11006667; doi:10.1038/s42003-024-06129-1)
Supplement: Supplementary file 2 — Description of Additional Supplementary Files [file 42003_2024_6129_MOESM2_ESM.pdf]

Description of Additional Supplementary Files

File name: Supplementary Data 1
Description: Cell lines from cfDNA panel in Figure 1E categorized by left (largest peak at ~167bp) or right skewing (largest peak at >1000bp) cfDNA release pattern.

File name: Supplementary Data 2
Description: Correlation of expression (TPM) from CCLE of DNASE family members with cell free DNA release and skew in various cell lines.

File name: Supplementary Data 3
Description: Correlation of expression (TPM) from Genentech dataset of DNASE family members with cell free DNA release and skew in various cell lines.

File name: Supplementary Data 4
Description: Commonalities exhibited by hits derived from MCF-10A CRISPR screen for regulators of cfDNA release at 0.5 Beta difference cutoff.

File name: Supplementary Data 5
Description: Commonalities exhibited by hits derived from A549 CRISPR screen for regulators of cfDNA release at 0.95 Beta difference cutoff.

File name: Supplementary Data 6
Description: Resource list for studies carried out in this manuscript.

File name: Supplementary Data 7
Description: The source data behind graphs in this paper.
