# Supplementary material for: An in vitro CRISPR screen of cell-free DNA identifies apoptosis as the primary mediator of cell-free DNA release
Source: Commun Biol. 2024 Apr 10;7:441. doi: 10.1038/s42003-024-06129-1 (PMC11006667; doi:10.1038/s42003-024-06129-1)
Supplement: Supplementary file 5 — Reporting Summary [file 42003_2024_6129_MOESM5_ESM.pdf]

nature portfolio

Corresponding author(s): Ben Ho Park

Last updated by author(s): Mar 14, 2024

Reporting Summary

Nature Portfolio wishes to improve the reproducibility of the work that we publish. This form provides structure for consistency and transparency in reporting. For further information on Nature Portfolio policies, see our Editorial Policies and the Editorial Policy Checklist.

Please do not complete any field with "not applicable" or n/a. Refer to the help text for what text to use if an item is not relevant to your study.
For final submission: please carefully check your responses for accuracy; you will not be able to make changes later.

Statistics

For all statistical analyses, confirm that the following items are present in the figure legend, table legend, main text, or Methods section.

n/a	Confirmed	
☐	☒	The exact sample size (*n*) for each experimental group/condition, given as a discrete number and unit of measurement
☐	☒	A statement on whether measurements were taken from distinct samples or whether the same sample was measured repeatedly
☐	☒	The statistical test(s) used AND whether they are one- or two-sided<br>*Only common tests should be described solely by name; describe more complex techniques in the Methods section.*
☐	☒	A description of all covariates tested
☐	☒	A description of any assumptions or corrections, such as tests of normality and adjustment for multiple comparisons
☐	☒	A full description of the statistical parameters including central tendency (e.g. means) or other basic estimates (e.g. regression coefficient) AND variation (e.g. standard deviation) or associated estimates of uncertainty (e.g. confidence intervals)
☐	☒	For null hypothesis testing, the test statistic (e.g. *F*, *t*, *r*) with confidence intervals, effect sizes, degrees of freedom and *P* value noted<br>*Give P values as exact values whenever suitable.*
☒	☐	For Bayesian analysis, information on the choice of priors and Markov chain Monte Carlo settings
☒	☐	For hierarchical and complex designs, identification of the appropriate level for tests and full reporting of outcomes
☐	☒	Estimates of effect sizes (e.g. Cohen's *d*, Pearson's *r*), indicating how they were calculated

Our web collection on statistics for biologists contains articles on many of the points above.

Software and code

Policy information about availability of computer code

Data collection	No software was used.
Data analysis	MaGECK-VISPR Version 0.5.6 was used to analyze the CRISPR Screen.

For manuscripts utilizing custom algorithms or software that are central to the research but not yet described in published literature, software must be made available to editors and reviewers. We strongly encourage code deposition in a community repository (e.g. GitHub). See the Nature Portfolio guidelines for submitting code & software for further information.

Data

Policy information about availability of data

All manuscripts must include a data availability statement. This statement should provide the following information, where applicable:

- Accession codes, unique identifiers, or web links for publicly available datasets
- A description of any restrictions on data availability
- For clinical datasets or third party data, please ensure that the statement adheres to our policy

Next-generation sequencing data generated for our CRISPR screen has been deposited at Dryad, and will be publicly available as of the date of publication (doi:10.5061/dryad.k0p2ngfd2), also listed in Supplementary Table 6. Source data for bar graphs can be found as Supplementary Data. Original western blot images are included in the supplementary figures. This paper does not report original code. All original CRISPR cell lines generated for this paper can be requested from the corresponding author. All other lines can be found through ATCC. Any additional information required to reanalyze the data reported in this work paper is available from the lead contact, Ben Ho Park (ben.h.park@vumc.org).

Research involving human participants, their data, or biological material

Policy information about studies with human participants or human data. See also policy information about sex, gender (identity/presentation), and sexual orientation and race, ethnicity and racism.

Reporting on sex and gender	N/A, no human subjects studied.
Reporting on race, ethnicity, or other socially relevant groupings	N/A, no human subjects studied
Population characteristics	N/A, no human subjects studied
Recruitment	N/A, no human subjects studied
Ethics oversight	N/A, no human subjects studied

Note that full information on the approval of the study protocol must also be provided in the manuscript.

Field-specific reporting

Please select the one below that is the best fit for your research. If you are not sure, read the appropriate sections before making your selection.

☒ Life sciences ☐ Behavioural & social sciences ☐ Ecological, evolutionary & environmental sciences

For a reference copy of the document with all sections, see nature.com/documents/nr-reporting-summary-flat.pdf

Life sciences study design

All studies must disclose on these points even when the disclosure is negative.

Sample size	Sample sizes were chosen before experiments as a balance between workload and having the highest n possible. For large experiments, such as our cell-free DNA panel, an n of three was elected because anything higher would have led to over 100 plates needing to be managed at once, which would have sacrificed the quality of the work on those samples for greater sample size.
Data exclusions	Some data were excluded in our correlation studies from our cell-line panel (5A, 5B). Exclusions are called out in the figure legend and methods. These data were excluded due to being outliers as measured by ROUT testing through Graphpad Prism at the most stringent level available. These removed samples were far and away different in one of the compared/correlated values from the main body of the samples, thus skewing any results we tried to draw if left in. All other datasets have had no exclusions.
Replication	All experiments were replicated at least once, run at an entirely different time. Results were consistent across trials in terms of significance, although magnitude of effect (ex. 2X increase vs 3X increase) was sometimes variable.
Randomization	We did not perform any experiments that required this given that our work is in vitro.
Blinding	We did not perform any experiments that required this given that our work is in vitro.

Reporting for specific materials, systems and methods

We require information from authors about some types of materials, experimental systems and methods used in many studies. Here, indicate whether each material, system or method listed is relevant to your study. If you are not sure if a list item applies to your research, read the appropriate section before selecting a response.

Materials & experimental systems

n/a	Involved in the study	
☐	☒	Antibodies
☐	☒	Eukaryotic cell lines
☒	☐	Palaeontology and archaeology
☒	☐	Animals and other organisms
☒	☐	Clinical data
☒	☐	Dual use research of concern
☒	☐	Plants

Methods

n/a	Involved in the study	
☒	☐	ChIP-seq
☒	☐	Flow cytometry
☒	☐	MRI-based neuroimaging

Antibodies

Antibodies used	Lamin A/C (1:1000, Cell Signaling Technologies, 2032), α-tubulin (1:1000, Abcam, ab4074), Sam68 (1:500, Santa Cruz, sc-1238), FADD (1:1000, Abcam, ab108601), GAPDH (1:1000, Cell Signaling Technologies, 5174), BCL-XL (1:1000, Cell Signaling Technologies, 2764), Goat anti-Rabbit IgG (H+L) Alexa 447 Fluor Plus (1:10,000 ThermoFisher, A32733), Goat anti-Rabbit IgG (H+L) Alexa Fluor Plus 488 (1:10,000, ThermoFisher, A32731), and Goat anti-Mouse IgG (H+L) Alexa Fluor 488 (1:10,000, ThermoFisher, A11029).
Validation	Multiple of these antibodies were used in this study to validate genetically proven knockouts, and their agreement with our genetic information validates them. CST2032: website shows validation westerns and lists important publications - Gruenbaum, Y. et al. (2000) J Struct Biol 129, 313-23 as an example. Ab4074: website shows validation westerns and lists important publications - Cooper-Knock J et al. Atypical TDP-43 protein expression in an ALS pedigree carrying a p.Y374X truncation mutation in TARDBP. Brain Pathol 33:e13104 (2023) as an example. Sc-1238: website shows validation westerns and lists important publications - Chen, T., et al. 1999. A role for the GSG domain in localizing Sam 68 to novel nuclear structures in cancer cell lines. Mol. Biol. Cell 10: 3015-3033 as an example. ab108601: website displays validation westerns and provides relevant publications - Zhou R et al. Clinical Impact of 11q13.3 Amplification on Immune Cell Infiltration and Prognosis in Breast Cancer. Int J Gen Med 15:4037-4052 (2022) as an example. CST5174: website displays validation westerns and relevant publications - Barber, R.D. et al. (2005) Physiol. Genomics 21, 389-95 as an example. CST2764: website shows validation westerns and lists relevant publications - Adams, J.M. and Cory, S. (1998) Science 281, 1322-6 as an example.

Eukaryotic cell lines

Policy information about cell lines and Sex and Gender in Research

Cell line source(s)	ATCC: MCF-10A, MCF-7, T-47D, BT-474, ZR-75-1, MDA-MB-231, HCT116, DLD-1. Lab of Dr. Emily Hodges at Vanderbilt: HEPG2/C3A, HEK-293T. Lab of Dr. Brian Lehman at Vanderbilt: SUM185-PE, MDA-MB-453, MDA-MB-468, HCC38, HCC70, HCC1143, HCC1937, HCC1806. Lab of Christine Lovly at Vanderbilt: A549, PC9, NCI-H841, NCI-H1607, NCI-H2227.
Authentication	STR profiling was employed on each of the cell lines used upon their entry into our lab. Results came back as expected for each cell line.
Mycoplasma contamination	These cell lines were tested for mycoplasma infection routinely and were consistently negative.
Commonly misidentified lines (See ICLAC register)	No such cell lines found in the ICLAC register were used.

This checklist template is licensed under a Creative Commons Attribution 4.0 International License, which permits use, sharing, adaptation, distribution and reproduction in any medium or format, as long as you give appropriate credit to the original author(s) and the source, provide a link to the Creative Commons license, and indicate if changes were made. The images or other third party material in this article are included in the article's Creative Commons license, unless indicated otherwise in a credit line to the material. If material is not included in the article's Creative Commons license and your intended use is not permitted by statutory regulation or exceeds the permitted use, you will need to obtain permission directly from the copyright holder. To view a copy of this license, visit http://creativecommons.org/licenses/by/4.0/
